# Base-Calling Algorithm with Vocabulary (BCV) Method for Analyzing Population Sequencing Chromatograms

**DOI:** 10.1371/journal.pone.0054835

**Published:** 2013-01-28

**Authors:** Yuri S. Fantin, Alexey D. Neverov, Alexander V. Favorov, Maria V. Alvarez-Figueroa, Svetlana I. Braslavskaya, Maria A. Gordukova, Inga V. Karandashova, Konstantin V. Kuleshov, Anna I. Myznikova, Maya S. Polishchuk, Denis A. Reshetov, Yana A. Voiciehovskaya, Andrei A. Mironov, Vladimir P. Chulanov

**Affiliations:** 1 Federal State Institution of Science Central Research Institute of Epidemiology, Moscow, Russia; 2 Department of Bioengineering and Bioinformatics, Lomonosov Moscow State University, Moscow, Russia; 3 Department of Oncology, Division of Biostatistics and Bioinformatics, Johns Hopkins University School of Medicine, Baltimore, Maryland, United States of America; 4 State Research Institute of Genetics and Selection of Industrial Microorganisms GosNIIGenetika, Moscow, Russia; 5 Vavilov Institute of General Genetics, Russian Academy of Sciences, Moscow, Russia; 6 Engelhardt Institute of Molecular Biology Russian Academy of Sciences, Moscow, Russia; 7 Department of Statistics, University of California, Berkeley, California, United States of America; 8 Institute for Information Transmission Problems (the Kharkevich Institute), Moscow, Russia; Cornell University, United States of America

## Abstract

Sanger sequencing is a common method of reading DNA sequences. It is less expensive than high-throughput methods, and it is appropriate for numerous applications including molecular diagnostics. However, sequencing mixtures of similar DNA of pathogens with this method is challenging. This is important because most clinical samples contain such mixtures, rather than pure single strains. The traditional solution is to sequence selected clones of PCR products, a complicated, time-consuming, and expensive procedure. Here, we propose the base-calling with vocabulary (BCV) method that computationally deciphers Sanger chromatograms obtained from mixed DNA samples. The inputs to the BCV algorithm are a chromatogram and a dictionary of sequences that are similar to those we expect to obtain. We apply the base-calling function on a test dataset of chromatograms without ambiguous positions, as well as one with 3–14% sequence degeneracy. Furthermore, we use BCV to assemble a consensus sequence for an HIV genome fragment in a sample containing a mixture of viral DNA variants and to determine the positions of the indels. Finally, we detect drug-resistant *Mycobacterium tuberculosis* strains carrying frameshift mutations mixed with wild-type bacteria in the *pncA* gene, and roughly characterize bacterial communities in clinical samples by direct 16S rRNA sequencing.

## Introduction

The method of direct, or population, sequencing of PCR products is widely used in medical diagnostics and for scientific purposes. Chromatograms obtained by this method contain information about mixtures of DNA variants, which are simultaneously amplified by PCR. The challenge we address here is the extraction of information characterizing the genetic diversity of the DNA variants without expensive and/or laborious methodologies, including PCR product pre-cloning, Single Genome Sequencing (SGS) [1] or Ultra Deep Sequencing (UDS) [2], [3]. The applicability of the aforementioned methodologies for mixture deconvolution is still limited in clinics because of their cost and/or complexity. SGS and sequencing after cloning is time- consuming and costly because a large (>100) number of cycles of PCR/dilutions or clones must be sequenced to detect a minor variant (about 1–2% fraction) with high confidence (>95%) [4]–[6]. In some cases, the sequencing of even 25 clones is insufficient to outperform the detection limit of direct sequencing assays [6]. UDS is a new powerful method that could be used for detection of mutations occurring in less than 1% of a mixture. However, many questions need to be answered before this method can be widely used in clinics [7], [8], and UDS is still at least four times more expensive and three times more time consuming per sample than direct sequencing [9].Currently, UDS is cost effective only if the device is completely loaded at each run [10].

For many clinical studies, direct sequencing is recommended [4], [11], [12] as an effective and inexpensive method for monitoring drug resistance. Many direct sequencing assays are commercially available for the mutation analysis for HIV-1 (Invitro Diagnostics Assays: e.g. ViroSeq HIV-1 Genotyping System, Abbott, TRUGENE® HIV -1 Genotyping Assay, SIEMENS), HCV (Research Use Only Assays: e.g. Virco, Janssen Diagnostics BVBA) and HBV (Research Use Only Assays: e.g. TRUGENE® HBV Genotyping Assay, SIEMENS). Direct sequencing is considered the gold standard for HCV subtyping [13].

### Difficulties

Direct sequencing is rather insensitive for minor DNA fractions that carry single-nucleotide substitutions (20–25% [3], [12], [14]); the sensitivity depends on the total DNA concentration in the sample [15]. Mixtures of significantly different DNA variants produce very complex chromatograms that are difficult to interpret with standard methods. Even when DNA variants are nearly identical, differing, for example, only by short insertions or deletions (indels), the chromatogram is still very complex.

All of these obstacles limit application of direct Sanger sequencing. It is not applicable for tasks like 16S rRNA gene sequencing for human specimens, determining HCV or HBV genotype in a case of mixed infection, and detection of indels.

### Existing Approaches

Original methods for reading cloned DNA from a chromatogram (e.g. Phred [16], [17]) allow for identifying only one type of DNA. Thus, these methods do not, in general, work for direct sequencing of a DNA mixture. Due to the active growth of resequencing projects, some tools for processing chromatograms of heterozygous genomes have been developed: e. g., TraceTuner [18] re-analyzes chromatograms (i. e., performs base-recalling) after the primary sequence has been defined. To avoid misinterpreting sequencing artifacts as single nucleotide polymorphisms (SNPs), a number of algorithms, like Polyphred [19], [20], Polybayes [21], and AutoEditor [22], require pre-assembled DNA sequences of various individual genomes. These algorithms are designed for resequencing eukaryotic genomes with a low degree of variability, and they require relatively high coverage of each genome site. More complicated is the identification of indels using direct sequencing data. Currently, some algorithms allow indel detection in heterozygous genomes[23]–[27].

Many of the problems concerning direct sequencing of heterogenous DNA sequences are summarized in [28]. A clinical sample may contain any number of DNA variants, with any mutation types (single nucleotide variations (SNVs), indels, and substring substitutions). In order to decipher a structure of the mixture, the wild-type sequence and the vocabulary of all allowed mutations must be provided. This formulation is a special case of the set-cover problem [29]. Even after this generalization, the algorithm is not universal: it is not feasible to choose a single wild-type sequence in rapidly evolving organisms such as RNA viruses. Also, it is impossible to choose one for the sequencing of 16S rRNA PCR products of a polyspecies sample. In many cases, it is also impossible to compile an exact vocabulary of allowed mutations, although the task of compiling a vocabulary of sequences homologous to those in the sample seems realistic. The RipSeq WEB server is an application designed for analysis of complex direct sequencing chromatograms, which are obtained for 16S rRNA PCR products from clinical samples and which accurately identify the bacterial species [30]. However, it is not a universal application for sequence analysis because it can process only chromatograms that are read from specific sequencing primers. The software maps all possible short words on the {A,C,G,T} alphabet that can be extracted from a IUPAC [31] sequence of a chromatogram onto known 16S rRNA sequences from a vocabulary. The RipSeq software can detect the presence in the mixture of up to three species from a vocabulary, and it does not generate sequences at the output. Practical methods of indel detection in direct sequencing chromatograms without a known wild-type also exist [25], [27], [32]. The Indelligent tool [25] identifies the two most similar sequences containing deletions assuming heterozygota. The CHILD [27] aligns the primary and secondary sequences that were extracted from a chromatogram by Phred; the alignment process uses SSEARCH [33]. CHILD can detect indels in low fractions (5–10%) of DNA mixture. Other tools quantify the components of complex mixtures by analyzing direct-sequencing chromatograms [34], [35].

### The BCV Algorithm

In this study, we propose a new method for analyzing population sequencing chromatograms, called base-calling with vocabulary (BCV). This method provides a comprehensive toolbox for Sanger chromatogram analysis. As input, this software uses the sequence of detected peaks in the chromatogram and the multiple alignment of nucleotide sequences similar to the expected DNA variants in the sample. We refer to this set of nucleotide sequences as the vocabulary. We did not assume any restriction on the number of possible mixed DNA variants. The BCV package contains tools for 3 main functions:

- base-calling,
- indel detection relative to the main consensus sequence and vocabulary sequences, and
- DNA mixture deconvolution.

Base-calling is sufficient for samples containing SNVs as the major mutation type. BCV does not require a vocabulary in this case.

**Figure 1:**
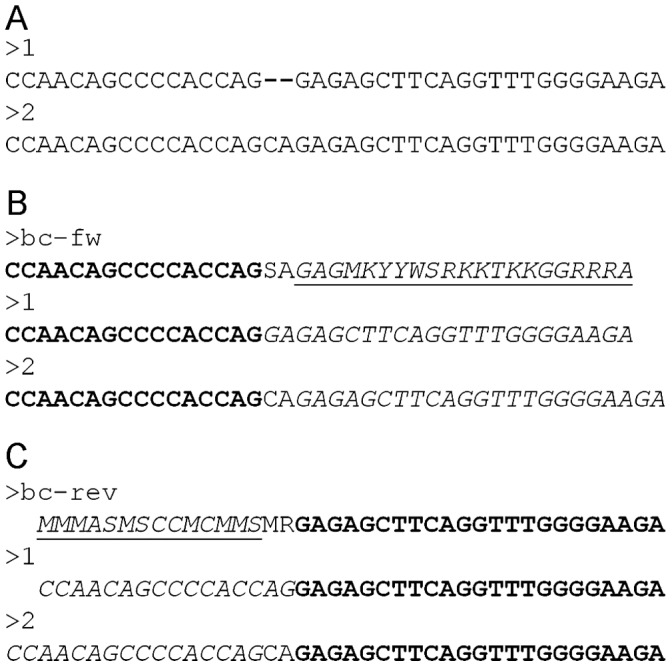
The shifting patterns in direct sequencing chromatograms. A. Sequences of 2 mixed DNA types (1 and 2) and their alignment. B and C. Chromatogram sequences (results of base-calling) for both reading directions are named bc-fw and bc-rev. The final Indels are assigned relatively to the main subgroup that is comprised of the DNA type, which has the higher fraction in the mixture. Italic underlined font shows shifting patterns. Bold shows the sequence portions that precede indel positions in each reading direction. Italics show the sequence portions of 2 DNA types that are aligned with the given coordinate shift.

The indel detection function is appropriate for analyzing chromatograms with a high proportion of degenerate positions when some of the sample variants carry indels (Fig. 1). A high homology of DNA variants is necessary for using the indel detection functionality. Otherwise, the most general functionality should be preferred.

The most general functionality of the BCV package is the mixture deconvolution. The software can predict sequences of DNA variants that altogether explain the amplitude profile of the chromatogram, provided that a vocabulary of sequences that are similar to actual mixture components is available. This functionality is effective only if the vocabulary contains sequences that are more similar to mixture components than the mixture components are similar to each other. When BCV is used for the mixture deconvolution, the predicted DNA variants can be further explored by Blast [36] homology searches in biological sequence databases, or by a kind of phylogenetic analysis. This functionality can be applied to complex mixtures of DNA variants, like direct sequences of 16S rRNA genes from clinical samples, and for detection of viral genotypes (e.g. HCV, HBV, HIV) by sequencing assays in the cases of mixed infections. Table 1 summarizes empirical rules for selecting the appropriate BCV functionality for a given sample.

**Table 1:** The guide for selection of the BCV usecase.

Expected diversity (eDiv)	Prevailed mutation type	Vocabulary not available (evDist>eDiv)	Approximate Vocabulary (evDist≈eDiv)	Representative Vocabulary (evDist<eDiv)
≤10%	SNV	Basecalling	Basecalling, Indel detection	Basecalling, Indel detection, Deconvolution
>10%	SNV and/or indels	Basecalling	Basecalling	Basecalling, Deconvolution

The table explains empirical rules that could be used for choosing appropriate BCV usecese depending on the expected diversity of DNA variants in a sample study. Expected diversity (eDiv) is the mean divergence of DNA variants expected in the sample. Prevailed mutation type is the most frequent type of mutations expected for sequenced DNA locus. Expected vocabulary distance (evDist) is maximal identity for DNA variants in the mixture with the sequences in the vocabulary that we expect: e.g. for human genome we can expect evDist = 0.001, for HBV surface antigen evDist<0.03. The vocabulary is considered as approximate if evDist≈eDiv; we cannot deconvolute mixture of DNA variants but still can detect indels if components of the mixture are similar. The vocabulary is considered as representative if evDist<eDiv, enabling deconvolution of the mixture into sequences in which genotype could be determined by similarity searches or phylogenetic analysis. The threshold 10% on expected diversity is approximate value.

We consider sequencing chromatogram processing to consist of the following steps: peak detection, base-calling, mixture deconvolution, and indel detection. The peak detection step is required to extract information from the 4 raw fluorescent traces obtained by a sequencer. We use the TraceTuner [18] program to determine the primary nucleotide sequence, and the Polyscan [24] program for re-base-calling. Since the source code for PolyScan is available, we add an option to output all the detected peaks, their physical properties, and the corresponding peaks’ probabilities into text files (refer to [24] for further details); thus, we don’t use the PolyScan’s grouping of peaks into sequence positions.

For sequencing of mixed DNAs, a chromatogram and its peak interpretation (partitioning) is represented as a hidden Markov model (HMM; generalized from [37]). Each partitioning represents an assignment of sequence positions to peaks, filtering out artifact peaks. A DNA base occurs only once in a sequence position; thus, each position can be described by an IUPAC DNA code. The base-calling step is a search for an optimal partition by the Viterbi algorithm [38], [39] for HMM on the chromatogram’s peak sequence. The mixture deconvolution step is intended to determine the DNA variants of the mixture that produced optimal partitioning. The variants are constructed by a greedy method that is based on preliminary DNA variants production by pairwise alignments of the partition with vocabulary sequences. Alignment scores are set up according to the standard HMM pairwise alignment schema, weighted by the peaks’ amplitudes. At each step, the sequence is determined from the best alignment; then the peaks that correspond to the found sequences are lowered (by subtracting the sequence from the mixture). Then the greedy search is repeated. Finally, the greedy-predicted DNA variants are combined by expectation maximization (EM) clustering procedure [40]. The distance measure for a pair of sequences, which is used for clustering, is a monotonically increasing function (see Methods) of the probability that the pair has been obtained from different chromatogram profiles. Initial clustering is made by a procedure similar to DOTUR [41]. The term ‘DNA variant’ refers to the consensus sequence found by the EM algorithm, unless explicitly stated otherwise. Expectations for DNA variant frequencies are evaluated from the clustering.

In the indel detection step, we use another clustering paradigm. All chromatograms for a sample are analyzed together. We suppose that all the greedy-generated (preliminary) DNA variants are classified into dense subgroups: those containing indels (one group per indel), and the group without indels (main subgroup). In each group, the preliminary variants are aligned with each other and with the vocabulary. After the main subgroup is identified for each chromatogram, we search for shifting patterns. A shifting pattern is a fragment on the chromatogram sequence that corresponds to a segment on preliminary DNA variants and that is aligned without indels against the main subgroup (Figure 1). Indels we are looking for occur before the start of shifting patterns (towards a read direction). Indel positions are calculated relative to the vocabulary sequences. Then we combine all the chromatogram’s main groups into the main consensus sequence that corresponds to the dominating pool of DNA variants in the sample.

We demonstrate the applicability of BCV in several situations. First, we use base-calling for analyzing direct sequencing traces of the hepatitis A and D viruses. Second, we use BCV to detect indels in the *pncA* gene of *Mycobacterium tuberculosis*. Finally, we apply BCV to deciphering a sample containing 2 subtypes of the hepatitis B virus (HBV) and complex mixtures of bacterial 16S RNA in human gastric mucosa by using direct sequencing.

The *M. tuberculosis pcnA* gene encodes the enzyme pyrazinamidase, which converts the pro-drug pyrazinamide to its active form, pyrazinoic acid [42]. In a considerable portion of cases, pyrazinamide resistance in tuberculosis occurs due to mutations disrupting the open reading frame of the the *pncA* gene [43]–[45]. Mutations found in resistant strains include amino acid substitutions, nonsense mutations, frameshifts, mutations in the promoter region, and even complete deletions of the *pncA* gene [43], [44]. Currently, over 350 mutations associated with resistance to pyrazinamide have been identified [46]. Traditional methods of sequence analysis work for detecting pyrazinamide resistant strains of *M. tuberculosis* in samples, but often fail to identify the presence of resistant strains in samples also containing wild-type bacteria.

## Results

### Samples and Cultures

All patients signed an informed consent form in accordance with the institutional review board of the Federal State Institution of Science Central Research Institute of Epidemiology, Moscow. The study was approved by the institutional ethics committee.

#### *M. tuberculosis*

Clinical specimens were obtained from patients at the Moscow Tuberculosis Clinical Hospital #7. A total of 123 clinical specimens, including sputum and other respiratory specimens, and bodily fluids were examined. *M. tuberculosis* cultures were grown on a Lowenstein-Jensen solid medium, and pyrazinamide susceptibility testing was performed with the BACTEC MGIT 960 System (Becton, Dickinson & Co., Franklin Lakes, NJ, USA) according to the manufacturer’s instructions.

#### Human immunodeficiency virus (HIV)

A plasma sample (ID GEN014DR.01A) was obtained from the control panel GEN014DR conducting external quality assessment of laboratories performing genetic tests to identify HIV HAART drug resistance mutations (HANC VQA Proficiency Testing Program website http://www.hanc.info/labs/labresources/vqaResources/ptProgram/Pages/default.aspx Accessed 20 Dec. 2012). The sample contained HIV subtype B with a viral load value of 8045 copies/mL.

#### Hepatitis viruses. Hepatitis B virus (HBV)

Plasma sample BV1 was obtained from a patient with chronic hepatitis B. Additionally, two plasmids containing the full genomes of HBV subtypes F2 and D1 according to [47], [48] were used in this study. Plasmids were used to prepare a mixture (df7) of subtypes in a 1∶2 ratio. The total concentration of DNA in df7 was 10^4^ copies/mL.

#### Hepatitis A virus (HAV) and hepatitis D virus (HDV)

79 serum samples from HAV-infected patients and 39 serum samples from HDV-infected patients were employed in this study.

#### Gastric mucosa samples

We studied two gastric biopsies (identified as sample 95 and sample 97) from children (5 and 17 years old, respectively). For both samples, *Helicobacter pylori* PCR (Amplisens H. pylori-FL, CRIE, Russia) and Rapid Urease Test (AMA RUT, Association of Medicine and Analytics, Russia) were performed.

For the details see Methods S1.

### Indel Detection in Clinical Samples

**Figure 2:**
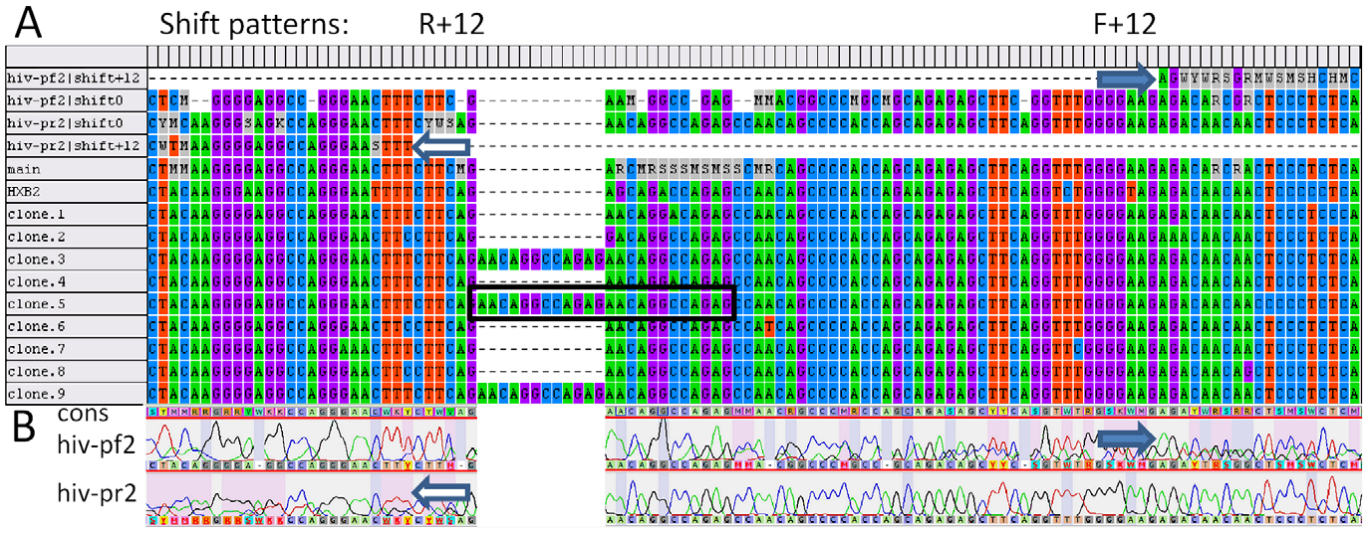
BCV-predicted shifting patterns and tandem repeat positions for the human immunodeficiency virus (HIV) sample GEN014DR.01A show the multiple alignment of sequenced clones and consensus sequences for shifting patterns including the main consensus sequence (A) and chromatogram trace images (B). Tandem repeats were highlighted by a frame on the sequence of clone 3. The beginning of shifting patterns, as for example for the hiv-pf2|shift +12 pattern, is marked by arrows. Sequencing primers are hiv-pf2 (forward) and hiv-pr2 (reverse). HXB2– is a reference sequence.

If DNA variants that contain indels were directly sequenced, the chromatogram was highly degenerate downstream from the indel sites (Figure 1). The results of the BCV indel detection analysis for the HIV, HBV, and *M. tuberculosis* clinical samples are shown in Table 2. Most samples in table 2 were obtained from our study (see below) of prevalence of *pncA* mutations in *M. tuberculosis*. Indel lengths varied from 1 to 12 nucleotides. For five samples, predictions made by the BCV were also confirmed by clone sequencing; for one sample the cloning experiment was not available (see Methods S1 and Table 2). The data produced by BCV indel detection analysis for the HIV sample GEN014DR.01A (Table 2) are shown in Figure 2. Sequencing the 3′ end of the HIV *gag* gene and the complete protease was done in both forward and reverse directions, using primers hiv-pf2 and hiv-pr2 correspondingly (see table S1). For the HIV sample GEN014DR.01A, the indel location was detected with minor error (−6 b.p) from the reverse sequencing primer, and the same indel location was detected with a larger error (+36) from the forward primer. The sample contained a mixture of HIV DNA variants; some of them carried tandem repeats relative to other strains that were in excess in the mixture. This error occurred due to some unrecognized minor peaks at the beginnings of the high polymorphism density regions of the chromatograms.

**Table 2:** Detected insertions and deletions (indels).

Organism	SampleID	Reads	Predicted Indel (type*,position)	Error in indel location
HIV	GEN014DR.01A	R	(0,+12), 2134	−6
		F	(0,+12), 2179	+39
*M. tuberculosis*	11042	F+R	(−6,0), pncA 298	0
		F+R	(0,+1), pncA 426	0
*M. tuberculosis*	ms41	F+R	(−1,0), pncA 89	0
	2243	F	(−2,0), pncA −3	n/a
	2687	F+R	(+1,0), pncA 325	0
HBV	BV1	2F	(−1,0), 924	0

Indel positions were annotated by sequencing selected clones of PCR products. Indel positions are accounted relative to the beginning of the RefSeq genome sequence or to the beginning of the *Mycobacterium tuberculosis pncA* gene. The number of reads supporting indel and their orientation (F/R) is shown. Error in indel location is the difference between a predicted indel position and the position of indel shown by clone sequences; “n/a” shows that the cloning experiment was not done for a sample.

* type: (major strain vs. wild-type, minor strain vs. wild-type). E.g.,(0,+1) means major ≡ wild-type, minor has a 1 bp insertion.

**Figure 3:**
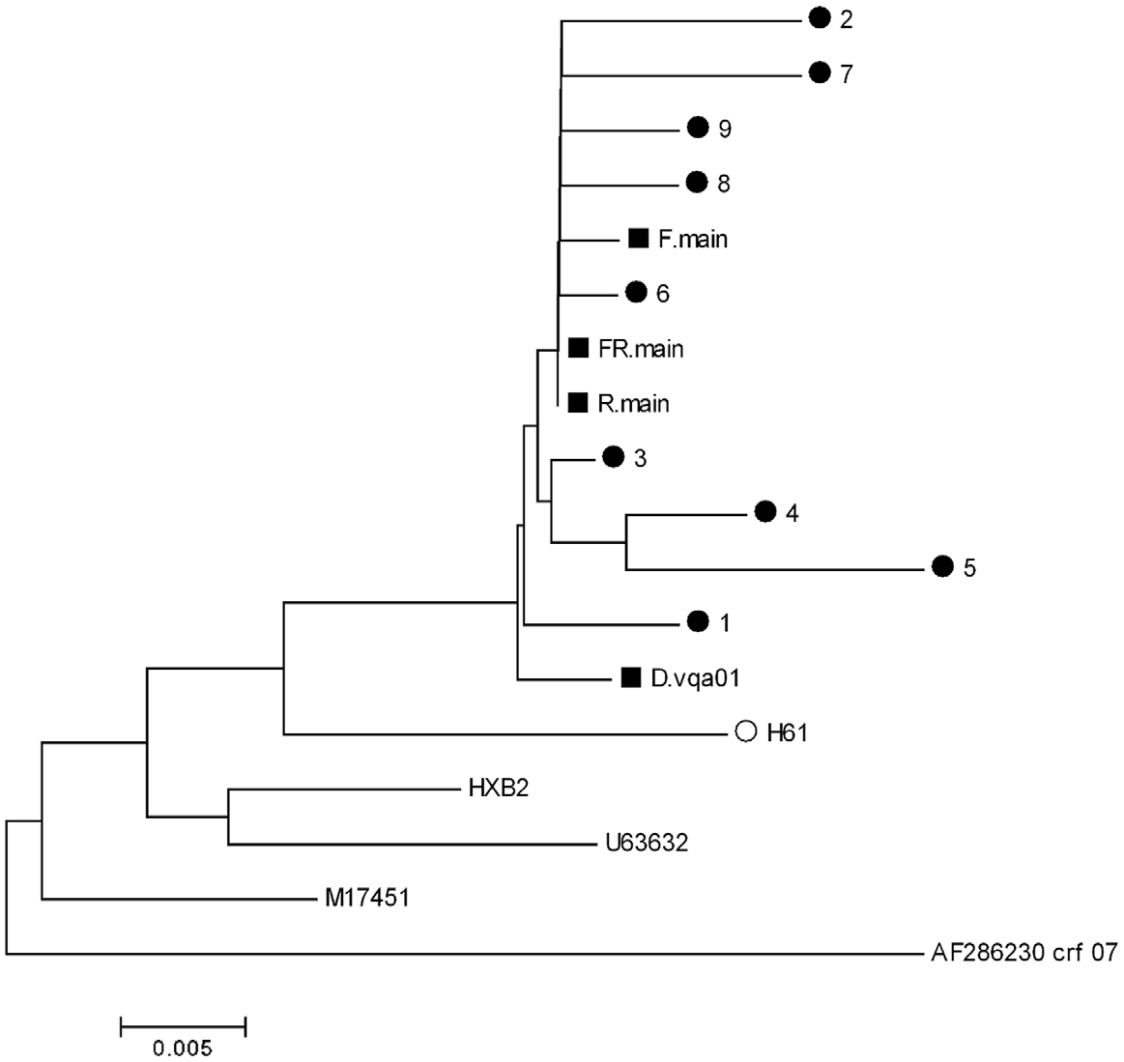
The comparison of BCV main sequence assembling results with sequences of cloned PCR products. Phylogenetic tree shows relationships between consensus sequences (black squares) assembled from direct reads of the HIV protease gene fragment with sequences of clones (black circles) for sample GEN014DR.01A. The consensus assembled from two opposite direct reads with trimmed degenerate parts is denoted as D.vqa01; the one that is assembled by the BCV indel detection script is FR.main. F.main is the dominating DNA type extracted from a direct read in the forward direction by the BCV indel detection script; R.main is the same read in the opposite direction. H61 is the blastn best hit to sequence D.vqa01 used for scaling quasispecies variation (black circles).Reads in forward and reverse directions have different fractions of non-degenerate positions: F: 56/503 = 11%; R –430/492 = 87%. B: a node in the tree corresponding to HIV subtype B branch. The phylogenetic tree is constructed by the Minimum Evolution method [66] for the Maximum Composite Likelihood [67] distance matrix by the MEGA 5 software [68].

We assessed how well the BCV indel detection script could determine the consensus sequence corresponding to a mixture of DNA variants containing indels. The BCV built a main consensus sequence that represented a pool of DNA variants that did not carry indels relative to each other. Preferably, the pool constituted the major portion of the mixture (main subgroup); in the case of an inability to select a subgroup of variants that clearly dominated in the mixture, the main subgroup was defined as the subgroup of variants with higher similarity to vocabulary sequences (see Methods). The phylogenetic tree that was shown at the Figure 3 had the following leaves:

- clone sequences (black circles)
- the consensus sequence obtained in the traditional way by assembling two sequences (reads); high density polymorphism areas corresponding to shifting patterns were trimmed (named D.vqa01)
- the main consensus sequences predicted for the hiv-pf2 and hiv-pr2 chromatograms by separate (F.main, R.main) and simultaneous analysis (FR. main)
- the closest homologue found by Blastn [36] search in GenBank [49] of clone sequences (H61-white circle)
- the sequence from the BCV vocabulary, which was used for predicting mixture content (HXB2)
- other reference sequences, used to define the distance variation within HIV subtype B

A high degree of heterogeneity was found in HIV quasi-species in the given sample (black circles). Consensus sequences (black squares) were located in the tree within quasi-species heterogeneity range around the best blastn hit H61. We wanted to emphasize that approximately 90% of the chromatogram derived with the forward primer (hiv-pf2 ) was degenerate. Thus, the algorithm was able to restore with reasonable accuracy the dominant DNA variant on a chromatogram with a highly degenerate sequence.

#### Prevalence of pncA mutations in *M. tuberculosis* strains isolated from clinical samples

The BCV was used to study *pncA* mutation prevalence *in M. tuberculosis* samples. We studied 123 samples that were tuberculosis-positive by microscopy. Five of the samples were negative for the *pncA* gene by PCR analysis, but were positive for another *M. tuberculosis* genome locus. The *pncA* gene was completely deleted in four of these PCR-negative isolates (3% of total samples), and one sample (0.8%) had an insertion of the mobile element IS6110, widespread in *M. tuberculosis* complex group [50], [51]. Among the 118 samples that were positive for the *pncA* gene by PCR, 54 samples (44%) contained only the wild-type *M. tuberculosis* strain. Of the remaining samples, 51 (41%) contained *pncA* mutants carrying amino acid substitutions. In eight of these samples, a wild-type strain was detected along with the mutant (with one or more SNV). *M. tuberculosis* strains isolated from 10 (8%) samples carried frameshift insertions in the *pncA* gene; two of these samples (1.6% of total dataset) were a mixture of mutant and wild type strains. Three isolates had *pncA* deletions, and two of them (1.6% of the common set) were detected in a mixture with the wild type. One sample (ID 11042; Table 2) was counted twice since it contained a mixture of strains with a 6 bp deletion in the *pncA* gene, strains with a 1 bp insertion, and wild-type strains. As a control, we also analyzed this sample by cloning and sequencing 10 selected clones, confirming the presence of indel-containing and wild-type strains in the sample. One sample (ID 2243) contained isolate with 2 bp deletion in position −3 upstream from the start codon in the mixture with the wild type.

Overall, four (Table 2) of the 123 clinical samples contained a mixture of wild-type strains and a mutated strain: three of them were with the *pncA* gene frameshift mutation and one with a *pncA* gene upstream indel. *M. tuberculosis* was isolated from these samples and was grown in cultures; pyrazinamid resistance was confirmed by direct phenotype tests on BACTEC MGIT 960 System. Indel positions were confirmed by sequencing of selected clones for three of these four samples.

#### Testing of the BCV detection limit and accuracy of indel predictions

The ability of the BCV package to detect indels of different sizes in minor DNA variants, which were presented in mixtures in different portions, was estimated using two test datasets, which were provided with CHILD [27] software. Both datasets contained chromatograms for mixtures of two clones of human mtDNA fragments with one of them having a deletion. The deletions’ sizes were 9 and 51 bp for the first and for the second datasets, respectively. For each fraction of minor variant, three chromatograms (replicates) were obtained.

BCV identified the presence of minor components carrying true deletions and components carrying indels of size 1 or 2 bp that also were detected in some mixtures by CHILD software. These indels were explained by the authors as artifacts of the cloning and sequencing procedure. For a moderate size deletion of 9 bp, the BCV was able to detect the presence of DNA variants with deletion in mixtures with minor variant portions of 10% or more (Table S3). Thus, BCV showed the same sensitivity as CHILD software on this dataset, but it was significantly more accurate in determination of the indel position. The error in estimation of the indel position was 1 bp for all samples in which the portion of the variant with deletion was 15% or more, whereas CHILD was less accurate, with indel start position varying by as much as 200 bp [27].

The BCV detection limit was estimated as 20% (Table S4) of the minor DNA variant carrying large 51 bp indel. The result was worse than that reported for CHILD, which was 5%. BCV predicted deletion of 52 bp for one of the three replicates with a fraction of 15%, and CHILD also erroneously estimated the size of deletion as 52 bp for three samples with a fraction of 20% or more of the variant with deletion. Also BCV failed to detect the variant with deletion in one replicate with a portion of 20%. The BCV exactly determined the positions of the deletion for all samples with fractions of 30% or more. The variation of the deletion coordinates was higher for CHILD than for BCV.

The study [27] also revealed the relative sensitivity and accuracy of indel size detection of CHILD with ShiftDetector [32] and Indelligent [25] software. BCV outperformed ShiftDetector and Indelligent in sensitivity as well as in specificity of the indel size detection; it demonstrated the same sensitivity and specificity as CHILD at a moderate size deletion dataset, and it was less sensitive and at least as specific as CHILD for a large deletion dataset. In any case, BCV demonstrated very good accuracy in detection of the indel position compared to CHILD.

We compared CHILD and BCV specificity for indel size prediction on clinical samples (Table 2), which were annotated by cloning and sequencing, so we knew the real sample sequences. CHILD correctly predicted the indel size for all samples except the sample “11042”. CHILD predicted 7 bp insertions in both forward and reverse chromatograms instead of the two 6 and 1 bp consecutive insertions relative to the dominating group of strains. BCV predicted both insertions correctly.

### Base-calling

The results of comparing base-calling accuracies of four applications (BCV, ABI base-caller [52], TraceTuner v. 3.01 [18], and PolyScan [24]) are shown in Table 3. BCV displayed an advantage over the other applications in specificity on the test datasets. The advantage reached 1% on the HAV dataset and 4% on the HDV dataset as compared with ABI base-caller, and 1% compared with PolyScan. The data models on which BCV and PolyScan are based allowed degeneracy of up to 4 peaks in each chromatogram position, unlike the other two applications. BCV and PolyScan were more specific on the HDV testing set of moderately degenerate sequences than ABI base-caller and TraceTuner (Table 3). All the programs showed a similar sensitivity exceeding 97% on both datasets. Table 3 shows the identities of predicted and annotated sequences. Identity statistics for BCV, ABI base-caller, and PolyScan were 89% on the HAV testing set. This value was significantly better than the identity obtained by TraceTuner. BCV and PolyScan had an advantage over the other two programs on the HDV testing set.

**Table 3:** Comparison of the base-calling accuracy statistics of Base-Caller with Vocabulary program (BCV) and other programs.

HAV				
	BCV	ABI Basecaller	Trace Tuner	PolyScan
Sn	0.97	0.98	0.98	0.98
Sp	0.90	0.89	0.86	0.89
ID	0.89	0.89	0.86	0.89
**HDV**				
	**BCV**	**ABI Basecaller**	**Trace Tuner**	**PolyScan**
Sn	0.98	0.98	0.97	0.98
Sp	0.91	0.87	0.84	0.90
ID	0.90	0.87	0.84	0.89

Sequences predicted by one of the following basecallers – BCV, ABI Basecaller 3100, TraceTuner v. 3.01 and PolyScan – are compared with manually assembled sequence datasets of genome fragments of Hepatitis A (HAV) and Hepatitis D (HDV) Viruses. The reference sequences in the HAV dataset do not have ambiguous IUPAC symbols and have moderate portion (3–14% ) of SNV in the HDV dataset. The standard measures of sensitivity, specificity and identity are shown.

### Mixture Deconvolution

#### Calculating the quality of correspondence between predicted and actual sample components

**Figure 4:**
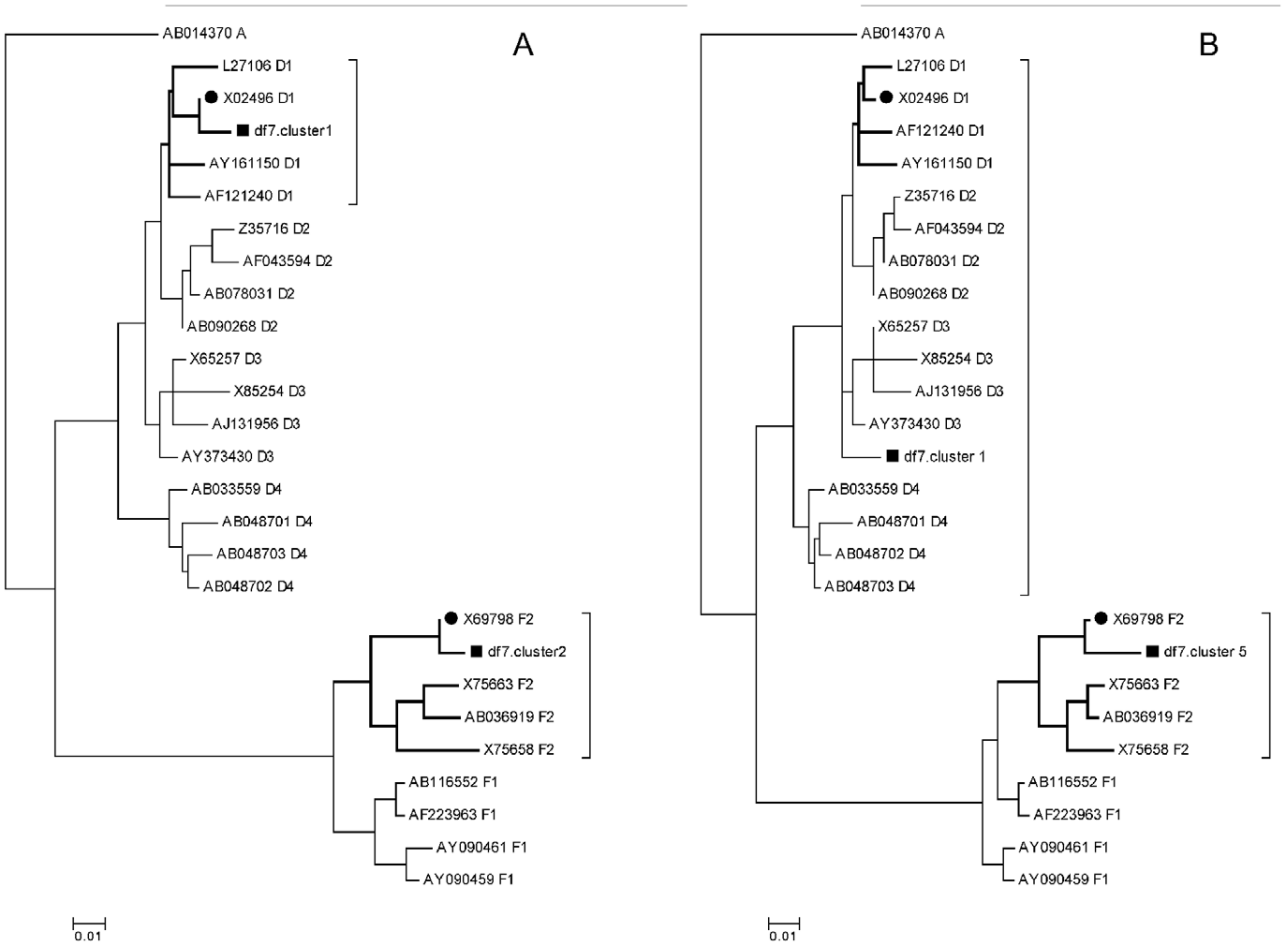
DNA types predicted by BCV for the sample composed from 2 components of D and F hepatitis B virus (HBV) genotypes. Black squares show predicted DNA types; black circles show actual sample components (identical to the GenBank sequences X02496, and X69798). Suffixes of sequence names correspond to HBV subtypes. Branches containing a mixture component are shown in bold. Right square brackets mark branches that contain predicted DNA types. The tics below the panels show the time scale. A and B correspond to two different vocabularies. A. Tree with DNA types predicted by BCV using the HBVRT vocabulary composed from 639 sequences of HBV genotypes A–H. B. Tree with DNA types predicted by BCV with vocabulary composed from 2 sequences approximately 0.028 substitution per site distant from components of the df7 sample. Phylogenetic trees are constructed by the Minimum Evolution method [66] for the Maximum Composite Likelihood [67] distance matrix by the MEGA 5 software [68].

**Figure 5:**
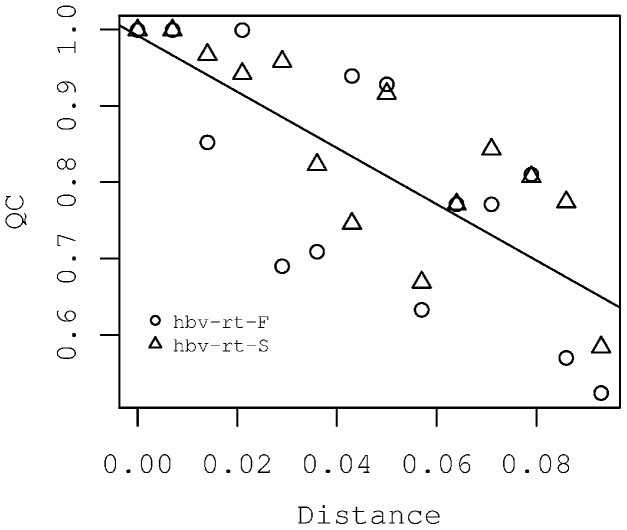
Dependence of mixture reconstruction accuracy on the level of similarity between vocabulary sequences and real components of the sample. The sample df7 that comprised a mixture of two HBV genome fragments of different genotypes (the same as on the figure 4) was sequenced from two primers “hbv-rt-F” and “hbv-rt-S” (see Table S1); each read was processed by the BCV using vocabularies of sequences that were on the different distances to the real mixture components. The Quality of Correspondence (QC) value of predicted and real components of the mixture is shown (see Methods S1).

A very relevant advantage of BCV over other methods of direct sequencing chromatogram analysis, which are based on subtraction of the reference sequence [23], [24], [28], [53], [54], is that BCV does not require an exact match between vocabulary sequences and actual sample components, and that, moreover, the number of variant DNAs does not need to be known. We assessed the effects of maximal distance (the number of differences) between vocabulary sequences and the actual DNA variants on the Quality of Correspondence (QC), which was defined as a measure of the relation of predicted DNA variants to the real components of the mixture. The QC measure was calculated on a phylogenetic tree that also contained both predicted, actual DNA types and vocabulary sequences. If the QC = 0, none of the predicted variants is situated within a subtree that grew from the most recent common ancestor (MRCA) of actual mixture components if QC = 1, all predicted sequences are placed in the subtrees of sister branches of actual DNA variants (see Methods S1 and Figure S1). For this, a model experiment was prepared. We directly sequenced a revertase gene fragment from a 1∶2 mixture of two complete HBV genome plasmids (F2 and D1 subtypes) using two primers “hbv-rt-F” and “hbv-rt-S”, and then used phylogenetic analysis to compare the predicted and actual DNA sequences as described in (Methods S1). Figure 4 shows two phylogenetic trees in which the predicted BCV DNA variants are located at different distances from the corresponding sample components. For clarity, only a small subset of the vocabulary is shown. The black squares in Figure 4A mark the sequences predicted by BCV using the complete vocabulary HBVRT (see Methods). Each of two predicted sequences belonged to the same subtype of HBV as the corresponding actual DNA (black circles). The sequences (black squares) in Figure 4B were predicted using a dictionary containing only two sequences; both of the sequences were an approximate distance of 0.028 substitutions per site from real sample components. One predicted sequence belongs to the same genotype as the corresponding mixture component, and the other belongs to the same subtype. The genotypes also included the actual corresponding sequences (black circles). Figure 5 is a plot of QC versus the maximal distance between vocabulary sequences and the actual DNA variants for two reads. The QC exceeded 0.85 in the [0, 0.028] distance range that roughly corresponds to intra-subtype variations of the HBV revertase gene. The distance between sample components F2 and D1 was 0.1. For both reads, the QC value decreased slowly from 1.0 to the 0.5 and its variation increased with distance.

#### 16S rRNA analysis of clinical sample microbial communities

**Figure 6:**
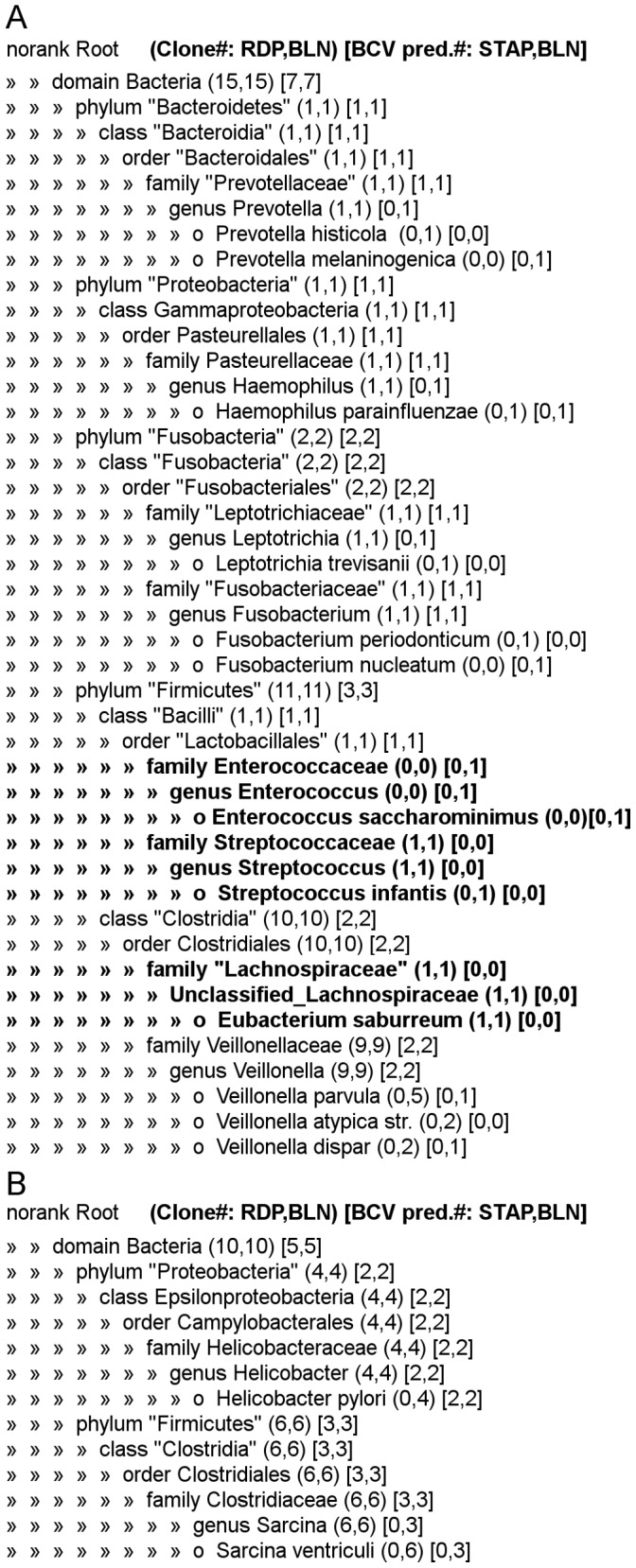
Comparing classification of DNA sequences of sequenced clones and BCV predictions of the 16S rRNA PCR product from a gastric mucosa biopsy. Each line corresponds to a single taxonomic category. Parentheses contain the number of sequences of clones classified using the RDP Classifier (first value) and the number of best alignments using blastn on the 16S rRNA database Greengenes (second value); brackets contain the number of BCV predictions classified by the method based on STAP (first value) and the number of best alignments using blastn on the 16S rRNA database Greengenes unambiguously assigned to that category (second value, see Table S2). Taxonomic tree represents the RDP classification. The species names of the best blastn hits are marked with circles. Inconsistencies in categorization between BCV and cloning are shown in bold. A. Sample 95. B. Sample97.

Figure 6 and Table S2 presented the results of taxonomical assignment of microbial populations from human gastric mucosa clinical samples, based on 16S rRNA analysis by two different methods: cloning and subsequent sequencing and classification using RDP classifier [55], [56], and direct sequencing followed by analysis by BCV, filtering out of rare (<5%) or short (<50% of chromatogram sequence length) predictions and classification using STAP (see Methods S1). For both types of analysis, sequences were searched by blastn [36] against the rRNA Greengenes database [57]. Figure 6 shows that the diversity of bacteria obtained by sequencing a small number of clones (10–15) coincided well with the results of direct sequencing followed by BCV analysis of three chromatograms per each sample. Table S2 shows the results of the search of the predicted sequences in the 16S rRNA Greengenes database. blastn hits with different taxonomy assignments were shown for a sequence if the assignment scores differed by not more than 2 bits. The similarity between predicted sequences and database sequences was within the 84–99% interval (the median is 95%); 9 of 12 BCV predicted sequences had identities with the best blast hit that were higher than 90%. All sequences except one were assigned by the STAP method into families or into more special categories - genera or species (table S2). Blastn was a suitable method to refine the STAP classification of predicted sequences, as hits had sufficiently high identities and had no contradictions with STAP (see Methods S1 and Figure S2 about the tolerance to errors of these classification methods).

There was a good correspondence between bacterial community characterizations by two methods: sequencing after cloning and direct sequencing followed by the BCV analysis. There was a (7/10) correspondence at the family level and a (7/10) at the genus level (figure 6). Two of three discordant taxonomy categories comprised only clone sequences: g. *Streptococcus* and *Unclassified_Lachnospiraceae* group, and one category (family *Enterococcaceae*) comprised a predicted sequence only. Contradictions between two methods could be explained at least in part by a small number of selected clones: e.g. five genera were observed only by one clone sequence per genus, and it was just by chance that these genera had not been missed.

Four 16S rRNA sequences obtained by cloning and two predicted by BCV in sample “97”, and no sequences in sample “95”, were classified into the *Helicobacter* genus (Figure 6). *H. pylori* was also detected in sample “97” by PCR and the rapid urease test; neither test detected *H. pylori* in sample “95”.

#### Comparison BCV vs RipSeq

The only software that we were aware of that could analyze the 16S rRNA gene sequencing chromatograms from clinical samples was RipSeq [30]. This software is a commercial Web server, and it processed only those chomatograms obtained using their own primers due to algorithmic features. We processed eight chromatograms available as usage examples (at RipSeq website https://www.ripseq.com/login/login.aspx Accessed 2012 Dec. 20) by BCV. The results are presented at Table S5 and are very similar to those provided by RipSeq.

## Discussion

Here we present new software, BCV, designed to analyze and decipher chromatograms of direct sequencing of mixtures of DNA variants. The procedure is based on a vocabulary compiled from sequences that are relatives to the mixture components, or, in more precise words, that represent all the available phylogenetic groups (genotypes or subtypes) of the organism under consideration, in order to achieve reliable detection of DNA variants that belong to these groups. Unlike other applications [24]–[28], [32], [35], BCV does not assume two components in the sample and does not need to know the exact wild-type sequence. The latter advantage is critical for numerous microorganisms and viruses.

Even for genomes with few mutations, direct sequencing chromatograms could have rather complex base-calling profiles due to indels, so the indel detection function of BCV is necessary for some clinical applications. The BCV is accurate both in detection of indel size and position (tables 2, S3 and S4), and it outperforms competing applications [25], [27], [32]. BCV allows for the execution of a joint analysis of multiple chromatograms, which are available for one sample, and thus it achieves a high accuracy of indel detection.

The BCV indel detection functionality also builds the main consensus sequence, relative to which the indels were detected. This sequence corresponds to a pool of DNA variants that includes most of the sample (or its sufficient part) and that can be aligned without deletions. When the reads are assembled in the traditional way, the highly degenerate parts of sequences have to be trimmed, decreasing coverage. We demonstrate good accuracy of BCV in assembling the main consensus sequence on an HIV sample containing a mixture of strains, some of which have deletions in the 3′-end of the gag gene (Figure 3). Accuracy of the three main consensus sequences (for forward and reverse directions and for both altogether) was estimated by a phylogenetic analysis. Each of the consensus sequences diverges from the clones to the same degree as the clones are diverged from each other. The main consensus sequence (see Methods for exact definition) could be used for genotyping of the target DNA.

We have shown the applicability of the BCV algorithm for assessing of rather complex DNA mixtures, such as bacterial populations by the direct 16S rRNA gene sequencing. The sequencing of 10–20 clones has a detection limit (10–20%) for minor DNA variants [4] that is similar with direct sequencing followed by BCV analysis. Thus, BCV could be a good alternative for cloning in some practical applications if a relatively high limit of detection of minor DNA variants is acceptable. BCV is not able to give very informative results for complex mixtures but could be very useful for characterizing the bacterial populations in clinical samples from body sites or liquids that are normally sterile [30].

The availability of a representative vocabulary for the mixture deconvolution is a key requirement for the success of the method. Figure 5 illustrates that the sequences from a vocabulary are the main source of information about linkage (correlations) of nucleotides in the predicted DNA variants. It is difficult to restore mixture components if the vocabulary sequences differ significantly from the component sequences because the variation of the amplitudes of some Sanger chromatogram peaks is too high to restore the correct nucleotide without any additional information. Thus, the mixture deconvolution functionality is recommended for those studies for which the goal is to test whether the known (or very close to the known) DNA variants occur in the mixture. Despite this requirement, BCV is sufficiently different from previously known applications that assumed identity between variants of DNA and vocabulary sequences [34], [35], while BCV assumes only similarity. Figure 5 shows that BCV produces valuable predictions when distances between the actual and the vocabulary sequences are below the divergence inside HBV subtypes (3%). The guide for selecting appropriate BCV functionality is presented in table 1.

RipSeq software [30] is also currently able to decipher 16S rRNA mixtures based on direct sequencing reaction. The algorithm, however, has several limitations: it can process only those chromatograms obtained from the proprietary RipSeq’s primers; and it doesn’t produce any sequences at the output, showing only match statistics for the RipSeq’s own pre-build vocabulary. Thus, it cannot detect presence of species that are distantly related to the vocabulary DNA variants. BCV is free of these restrictions. Furthermore, in contrast to RipSeq, a commercial web server, BCV is freely distributed in source codes and it produces sequences at the output. Thus, a researcher can iteratively extend the vocabulary used for BCV run based on the blastn search for predicted variants against a comprehensive database (e.g. Greengenes). If a prediction has a high BCV expectation to be a component of the mixture and it has a low identity in the blastn search it indicates a probable lack of the corresponding species in the BCV vocabulary. The mixture deconvolution procedure is then repeated after the missing sequence is included in the vocabulary. To address the question about the limit of minor variant detection by BCV, we used the chromatograms provided in the study [27] (tables S3 and S4). The limit of detection for BCV can be 10% of indel fraction for moderate size indels (<10 bp). For large indel size (51 bp), the higher detection limit (20%) is expected.

The SNV detection limit for the traditional method of direct sequencing chromatogram analysis was estimated at 20–25% [3], [12], [14] and highly depends on total template concentration [5]. Given that the main source of error in BCV DNA variants prediction is the loss of secondary peaks in the chromatogram sequences, we assume that, in general, the mixture deconvolution functionality could not be more sensitive to minor variants than the limit of detection for point mutations. This is because the accuracy of basecalling is a bottleneck for the following deconvolution step. Indeed, if only a few positions in the chromatogram distinguish different DNA variants and many artifact peaks have similar likelihood values, deconvoluting that mixture correctly is difficult. Some sequencing artifacts, like the shadow effect [27], [58], cannot be distinguished from true polymorphism; thus, variant calling is possible only if the amplitudes of the minor variant in true polymorphic sites are significantly higher than these artifacts.

In the cases of indel calling and deconvolution of highly heterogeneous mixtures, e.g. SSU rRNA genes that carry a lot of indels as common evolutionary changes [59], [60], BCV can improve sensitivity due to the excess of true polymorphic sites caused by shifts (Figure 1). On the one hand, indels greatly complicate the conventional analysis of direct sequencing chromatograms. On the other, they make mixture of homologous sites in one chromatogram position improbable, and thus the main source of error in predicted BCV DNA variants for 16S rRNA direct sequences is due to random substitutions at random sites rather than misinterpretation of homologous sites of different species as a variant inside a single species. For this reason, the more diverse a bacterial population is, the more DNA of the 16S rRNA types can be classified in it, since an alignment of 16S rRNA for more diverse species has a higher percentage of deletions. To achieve a better diagnostic sensitivity, we recommend making several reads of 16S rRNA gene for each sample and combining BCV predictions as done for gastric biopsy samples (see table S2).

BCV cannot be considered a universal alternative to the experimental gold standard methods (SGS, pre-cloning and sequencing, UDS) for estimation of diversity because BCV’s ability to detect minor DNA variants is limited by the sequencing method (Sanger). BCV can expand capabilities of population sequencing in various scientific and clinical studies, and it is an alternative to standard chromatogram analysis. For example, BCV cannot be used for deciphering of HIV quasispecies because it is impossible to provide a priori a representative vocabulary for such a mixture, but if such a vocabulary were obtained using one of the gold standard methods, it could then be used for monitoring quasispecies dynamics by BCV, thus saving time and money.

## Methods

### BCV Software Data Flow

**Figure 7:**
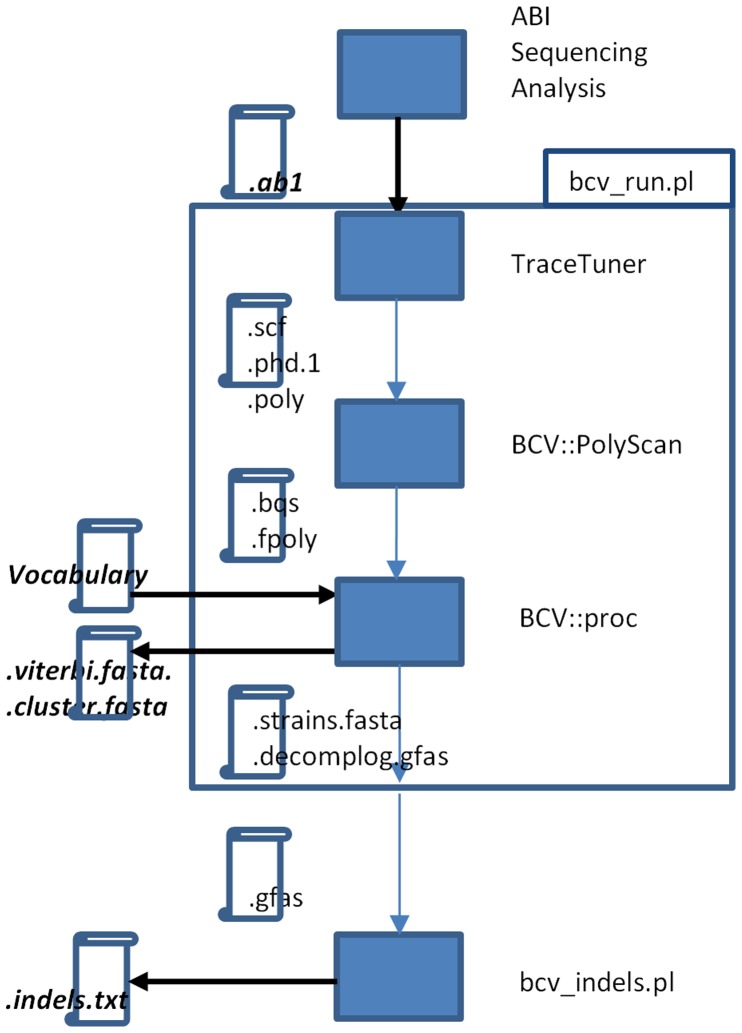
BCV dataflow. Rectangles depict software applications; rolls depict files; black arrows are the pipeline input and output streams with the corresponding input and output file extensions shown in italic bold. The file extensions are as follows: The input ABIF (*.ab1) file contains the chromatogram itself and the ABI base-calling. TraceTuner files (PHRED compatible): *.scf contains the chromatogram; *.phd.1 is the chromatogram sequence, and *.poly is the secondary peak calling results. PolyScan files: *.fpoly contains minor peak calls around the primary sequence, and *.bqs contains the peak likelihoods. BCV pipeline output files: *.viterbi.fasta contains the chromatogram sequence; *.cluster.fasta is the DNA type reconstruction and *.indels.txt is the indel report. The configuring and calling of TraceTuner, BCV::PolyScan and BCV::proc applications is enveloped in the bcv_run.pl script. For indel detection functionality the call of the bcv_indels.pl script is followed of the bcv_run.pl. The bcv_run.pl prepares an alignment of raw predicted DNA variants (from the *.strains.fasta file) with similar sequences from the vocabulary that are listed in the *.decomplog.gfas file. Both files are generated by the BCV::proc application. The input file for the indel detection script bcv_indels.pl has the grouped FASTA format and corresponding.gfas file name extension.

Figure 7 represents the data flow of the BCV software. The main BCV application BCV::proc receives tree input files: FPOLY file containing the peaks’ physical properties, BQS file containing the probability scores of the corresponding peaks in the positions and a vocabulary in FASTA format. The FPOLY and BQS files are generated by PolyScan [24] that received the primary sequence from TraceTuner [18] as input. The scores of the peak probability from the BQS file are used for trimming the 3′ artifacts at the end of the chromatogram; peaks in the 3′ tail with probability scores lower than 5% are excluded. Indel detection is done using the Perl script bcv_indels.pl. The script’s output (*.indel.txt) consists of shift patterns, the main consensus sequence, and the indel coordinates, which can be determined with respect to the main consensus sequence and to a specified vocabulary sequence. The script receives a multiple alignment of DNA variants in the GFAS format, obtained by the greedy deconvolution procedure. The GFAS format is similar to the FASTA format; it carries additional information ascribing sequences to chromatograms. Preparation of such an alignment is done by the Perl script bcv_run.pl. The script receives BCV::proc output files (*.strains.fasta and *.decomplog.gfas) for the same sample as the input. The dataflow is enveloped in the bcv_run.pl script that takes a path to a folder with ABI chromatogram files and a project XML file with specification of the correspondence of the chromatograms to samples and required functionality (usecase).

### BCV HMM Training

Parameters for the HMM of chromatogram base-calling were trained by a Baum-Welch algorithm [61] on a training set of 80 sequencing traces. For each trace in the set, an annotating sequence was available. The annotation was obtained by the manually curated assembling of two traces of HAV genome fragment 2C in both directions (NC_001489: positions 3796–4443). The training annotation sequences did not contain degenerate positions.

### BCV Vocabularies

The BCV application uses a multiple alignment of target genome fragments in FASTA format as the vocabulary for DNA mixture deconvolution. To analyze direct sequencing chromatograms of the HIV genome fragment containing the 3′ fragment of the *gag* and complete protease genes (K03455∶2052–2623), we created the vocabulary HIVPRT (HIV protease). The genome fragments were cut from whole genome sequences used in the NCBI Viral Genotyping Tool [62] for HIV-1 subtyping. Another vocabulary, HBVRT (HBV revertase), was used to analyze the revertase domain of the polymerase gene (X04615∶336–1161). It consisted of 669 sequences extracted from the complete HBV genome sequence alignment obtained from the HVDB [63] database, and assigned to 1of the 8 HBV genotypes (A–H). The vocabulary for analyzing the *M. tuberculosis pncA* gene contained one sequence of the corresponding genome fragment (NC_002755∶2291656–2290984). BCV vocabulary of human bacterial community 16S rRNA sequences was obtained from the Greengenes [57] server by merging two multiple alignments: HMP_strains_16S_aligned.fasta (Human Microbiom Project [64]) and human_assoc_gold_strains_gg16S_aligned.fasta.

### Algorithms

This chapter thoroughly describes the algorithms implemented in the BCV software.

### Main Definitions

#### Input data

D1 We will use the term *chromatogram* to denote a sequence of peaks: *Z = z_1_…z_N,_*, where.

the peak *z_i_* is a vector with the following components:

*x_i_* - the vector of the peak’s physical characteristics (height, width, etc.).

*t_i_* - the peak’s coordinate (on the gel),


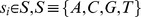
- the corresponding nucleotide.

*N* - chromatogram length (the total number of peaks).

Peaks in the chromatogram sequence are ordered by their coordinates: 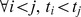
_._

D2 The *vocabulary:
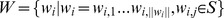
*.

#### Derivative model abstractions

D3 The *chromatogram sequence* is an extended (UIPAC) nucleotide sequence that is ascribed to the chromatogram by the *mapping operation*. The *mapping index* of peak *i* maps the chromatogram to the sequence: 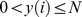; it is 0 for a false peak, or it is a position 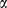
in the corresponding chromatogram sequence of generally ambiguous nucleotide codes (IUPAC). The monotony holds for the indices: 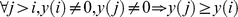


D4 The *chromatogram partition* 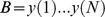 is a sequence of peak mapping indices. The length of the sequence is. There is a specific order on the values of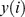, so the partition could be represented in the equivalent way as the sequence
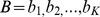
of *positional frames b_α_* for each chromatogram sequence position 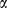.
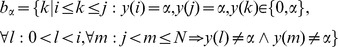



The *subset of true peak indices* in a positional frame *TP(b_α_)*

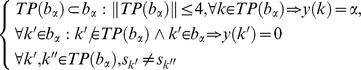



corresponds to an IUPAC symbol in position *α* in the base-called sequence.

D5 The term *DNA variant* is used to denote a DNA molecule present in the mixture. We assume that DNA variants are homologous and may correspond, depending on experiment, to viral quasispecies, bacterial strains, or homologous loci in a large genome. We define a sequence of indices that map the chromatogram peaks to the nucleotide sequence of a DNA variant as 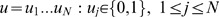. If index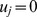, then the peak *z_j_* does not correspond to any nucleotide in the DNA variant *u*.

Only true peaks correspond to nucleotides in a DNA variant:
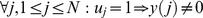



For each positional frame, there is one and only one true peak has to be selected.
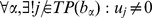



D6 The DNA *mixture* is a set of DNA variants.

D7 The vector of a mixture’s fraction components: *γ,
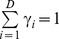
*_._

### Hidden Markov Model (HMM)

The expression for the probability of a chromatogram partition *B* is.


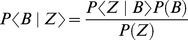
 *(I)*,

where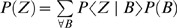. The task of chromatogram decomposition can be represented as the optimization of the functional (I):


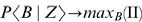
.

If we assume that the prior distribution *P(B)* is uniform, then the problem reduces to finding maximum likelihood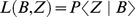. Likelihood is computed by an algorithm that generalizes the work of Andrade-Cetto (2005) [37] to the case allowing ambiguity in chromatogram sequence positions (the position’s degeneration). We assume that the correct peaks in positional frames are in first-order Markov dependence. We introduce an additional notation for true peak sets: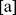. Letting 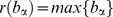
be the right-most true peak in the positional frame that is mapped to sequence position 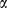, the operator […] assigns a sub-sequence, like a chromatogram sub-sequence
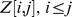
or a false peak sub-sequence 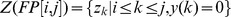
(sequencing artifacts in the [*i,j*] range of peaks). Then the likelihood of the chromatogram can be written as:
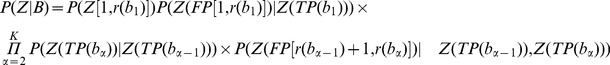
(III)


The first term on the right side of the expression is the initial probability of the Markov model as a product of two terms: one is concerning the *TP* peaks subset for the chromatogram start and the second is concerning the *FP* subset. The product on the second line accounts similar terms in a first order Markov chain manner.

The functional (III) can be considered as an HMM, so it can be optimized (II) by the Viterbi algorithm [38], [39].

#### True peak likelihood



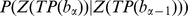



The sequence of true peaks in frame *α* can be written as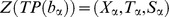. Applying the Bayes formula, and making a set of independence assumptions, e.g. assuming that independence of the coordinate in gel on the physical characteristics of the previous peak, we can rewrite the desired probability as follows:



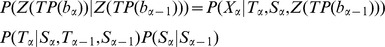



The last term of the expression is the *a priori* probability, which reflects our knowledge of the nucleotide composition of the genome locus. This probability can be expressed in terms of dinucleotide frequencies estimated from vocabulary *W*. If the peaks in the same positional frame are assumed to be independent of each other, then we obtain the expression: 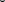. The evaluation of expression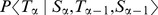 can be based on the assumption that peaks from different channels (A, C, G, T) appear in frame α independently of each other. Thus, we write: 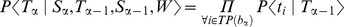; we calculate the probability of a peak to be true in a given positional frame as the peak’s most probable localization relative to true peaks in the preceding positional frame .

The likelihood for physical properties of true peaks in a positional frame 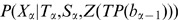
can be estimated on the basis of additional assumptions:

1. The dependence of a peak’s physical characteristics *X* in the positional frame on the coordinate vector *T_α_* can be defined as an interval function of *α* (quasi-homogeneous Markov model). We used the following intervals: the body 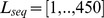 and the tail 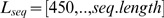. Dependency of *X* on *T_α_* is set by parameter *η*: .Within the intervals, for each peak, we assume independence between the peak’s characteristics *x* and coordinate *t,* as in [37]. If we assume such independence, then 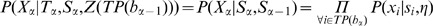.
2. In fact, a relationship exists between the gel coordinate of a peak and its amplitude: *x = I*. Peaks at the end of the chromatogram decrease in amplitude while their width increases. This dependence of peak amplitude of position is described in [65]. We write the intensity of the signal in channel *s*, corresponding to peak *j* in frame *α,* as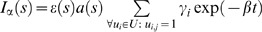, where the *ε(s)* is the noise in channel *s* with the corresponding probability density function: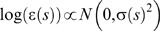 and summation is done over all DNA variants having a peak *j* in the frame *α*. Maximum amplitude in a channel *s* is expressed as *a(s)*. For a particular chromatogram, parameters *a(s)* and signal decay *β* can be estimated by the least square method at non-degenerated positional frames containing a single true peak. Another way to estimate these parameters is to use a partition generated by external software like PolyScan [24]. After normalization of peak amplitudes to expected amplitudes, the positional dependence can be removed: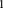. From here, *I* indicates normalized peak amplitude and *γ(s)* denotes the share of nucleotide *s* in the DNA mixture.

Finally, we obtain 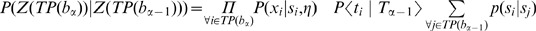.

#### False peak likelihood



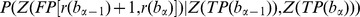
.

False peak probability decomposition is done similarly to true peak probability. Briefly, we denote 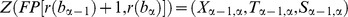, where vectors *X*, *T*, and *S* are a peak’s physical properties, coordinates, and symbols, respectively. Performing the Bayes decomposition and omitting the intermediate steps, we get 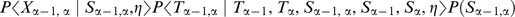. Here, vectors with double (e.g., *α-1, α*) and single (e.g., *α*) indices correspond to false and true peaks, respectively. Again we assume that physical properties *X* are independent of the coordinates *T* within intervals of a quasihomogeneous Markov model. To further simplify the expression, we associate the average true peak coordinates *t_α -1_* and *t_α_* in these positional frames with vectors *T_α-1_* and *T_α_.* We then express coordinates of false peaks from vector *T_α-1, α_* relative to the coordinates of the frames 
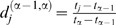
, where *t_j_* is a coordinate of the false peak in the chromatogram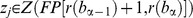.We assume that the probability 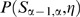
obeys a Poisson distribution to observe false peaks between positional frames *α-1* and *α*. Given the additional assumption of independence of false peaks, we finally write



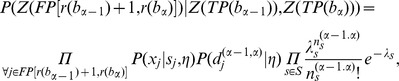
where


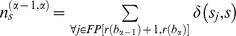
 is the number of false peaks between adjacent positional frames for each channel (A,C,G,T), and *λ_s_* is the expected number of false peaks.

### DNA Mixture Deconvolution Algorithm

Setting partition *B* as the optimal solution to task (II), we find a set of DNA variants (D6) that is the most likely to correspond to this partition. We align sequences *w* from vocabulary *W* against the partition. The algorithm of such an alignment is similar to an ordinary pairwise global alignment algorithm in the space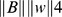. The third dimension corresponds to the nucleotide composition of the positions in the partition. We then choose a scheme of alignment weights proportional to relative nucleotide frequencies in positional frames, so for each sequence in the vocabulary, the best alignment will mostly pass through the highest peaks in frames that contain no peaks matched to these bases in the sequence. For each positional frame *α*, we determine the relative amplitude of each symbol 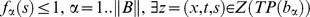. If a peak in this partition is treated as false, 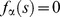. We represent relative amplitudes as array *F* with dimension equal to the number of peaks in the chromatogram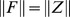. The mixture deconvolution algorithm determines a set of DNA variants 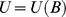. DNA variants will be represented by a sequence of elements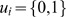, where 0 means that a peak does not emit a base for a DNA variant sequence. Below, we write the pseudo-code for the mixture deconvolution algorithm:

**Input.** (*Z*, *B*, *W*).

Parameters:

*K_max_* - the maximum number of components in the mixture, so *1/K_max_* is the minimum frequency with which the DNA variant may be detected. This frequency is determined by the minor variant’s detection limit of the sequencing method (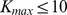).


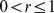
- rate of the amplitude descent. A reasonable value is 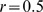.

**Output.** the DNA mixture *U = U(B)* and the vector of the mixture’s component fractions *γ.*

Calculations:

1. Calculate the peak’s relative amplitudes in positions. Set up the initial DNA variant fractions 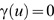.
2. Iterate for each sequence *w_k_* in vocabulary W
  2.1. Align sequence *w_k_* with (*B*, *S*, *F*). Alignment weight for a true peak corresponding to the symbol *s* in positional frame *α* is set to - 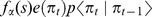, where *t* is an alignment position, and 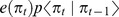
corresponds to emission and transition probabilities for a pairwise alignment HMM in state π_t_ = (match, opening an X insertion, opening an Y ins., X ins. elongation, Y ins. elongation, etc).
3. Take the alignment with the highest score. Set up elements for a new DNA variant *u_i_*: *u_i,j = _1* for each peak *j* used in the alignment, and *u_i,j = _0* otherwise.
4. Find peak *j’* with the minimal amplitude 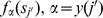
from all peaks used in the alignment.
5. For each peak where 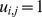, change the relative amplitudes to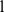.
6. Add new variant *u* into the hash table *U*. Update the variant’s fraction vector
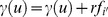

7. Terminate if there is a positional frame, where all peak frequencies are below the threshold - 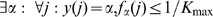, else go to 2.
8. Output (*U*, *γ*).

### DNA Variant Clustering

The sequences of DNA variants and their number depend on the parameters that are used for mixture deconvolution – *r*, *K_max_*. Since the sequence of each of the virtual DNA variants may contain various errors, it is important to combine similar DNA variants into clusters and to build consensus sequences for each cluster that decreases the error level in predicted sequences of DNA variants. Thus, the algorithm would make it possible to identify significantly different DNA variants by their genetic distance. To solve the clustering problem, we adapted the algorithm proposed in [40] for 454 reads.

We assume that the mixture deconvolution algorithm at each iteration *i* generates a unique variant. Consider a DNA variant 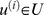
(here we used superscript indices to emphasize the parameters depended on an iteration number) and a nucleotide sequence *w* not necessarily belonging to vocabulary *W*. To use the algorithm proposed by Quince [40], we need to build a model of the distance between the DNA variant and the sequence 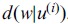. To consider a possible alternative choice of peaks in positional frames by the mixture deconvolution algorithm, we introduce a variant profile.



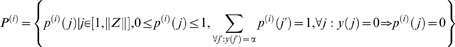
.

The variant profile assigns probability that each true peak emits a base for the variant *u^(i)^*. The profile represents an uncertainty model determining the chance that the peak is included into a variant sequence generated at iteration *i*. The distance between the variant and the sequence will be calculated under the condition that the profile is also known: 
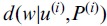
. We will further assume that we also have a pairwise global alignment of a DNA variant and a sequence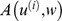, and, strictly speaking, the distance will be a function of the alignment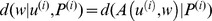. Here and below, unless otherwise noted, variant *u^(i)^* is regarded as a nucleotide sequence. Let us also set the base substitution model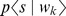 that determines the probability that base *w_k_* depends on base *s*. We then define distance.
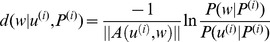
(1)through the probability of sequence *w* to be obtained from the profile of DNA variant *P^(i)^* and the substitution model. Expression
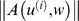
denotes the length of the pairwise alignment *ignoring indels*. Distance is normalized to alignment length in order to accommodate the different lengths of DNA variants. We now write an expression for calculating the probability that a sequence was derived from the specified variant profile:



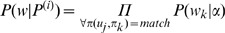
(2)Where



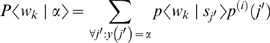
(3)


The sum in expression (3) is taken over all true peaks in a positional frame to which peak *j* belongs. The product in expression (2) is taken over all matches in the alignment of sequences *w* and *u^(i)^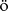.* Equation (1) can be rewritten as the sum of positional distances in the alignment of a DNA variant *u^(i)^* and sequence *w* using expression (2): Z



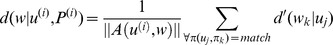
(1.1),

where



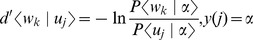
(1.2).

Probabilities
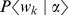
were calculated using expression (3). The final output of the expectation maximization algorithm described in [40] depends on the initial conditions. The initial variant grouping was done by the hierarchical clustering algorithm similar to the DOTUR [41]. We determine the distance between 2 DNA variants using the formula.



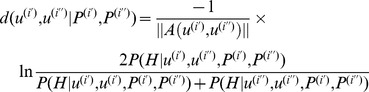
(4)where the expression
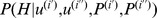
defines the probability of true homology between DNA variants *u′* and *u′′* (*i.e.,* the probability that both variants were derived from a single profile):



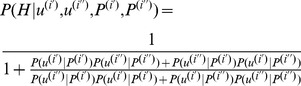
(5)Used in formula (4), the expression 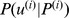
is evaluated in accordance with (2), assuming that *w = u^(i)^*.

Below we write a pseudo-code for the expectation maximization algorithm for clustering DNA variants:

**Input.** (*U*, {*P^(i)^*}).

**Output.** The set *C* of consensus sequences corresponding to set *U* of DNA variants.

Calculations:

1. Construct a multiple alignment of DNA variant nucleotide sequences. Denote 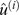 representing the nucleotide sequence of the DNA variant at *i-th* iteration of the mixture deconvolution algorithm in the multiple alignment. The length of this sequence is equal to the alignment length. In each sequence position, the following characters are valid: 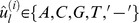.
2. Make initial associations of DNA variants into clusters. Pairwise distances between variants are calculated in accordance with expression (5). Pairwise alignments of variants are retrieved from the multiple alignment (column containing characters ‘-’ were removed). As a result, we obtain matrix **Z,** which is a correspondence table for variants and clusters. See [40] for details.
3. Iterate where matrix **Z** changes more than the specified accuracy threshold from one iteration to the next:
  3.1. The M-step: calculate the consensus nucleotide sequences for DNA variant clusters *U_j_* and their corresponding weights τ_j_. Expressions (6–8) use the following notations: *C^j^_l_* is a character at position *l* in the consensus sequence of cluster *j*; *K* is the number of DNA variants; *i* is a specified DNA variant; **z^i, j^** are elements of correspondence matrix Z; and distances *d′* are calculated according to expression (3). Consensus sequences are calculated taking into account the contributions of mixture fractions *γ* of DNA variants (see 8).

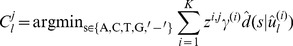
(6)

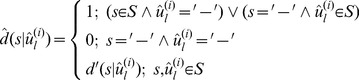
(7)

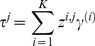
(8)
  3.2. The E-step: Calculating the expected values of elements **z^i, j^** of correspondence matrix Z. The expression for distances *d* was calculated in accordance with expression (1.1).

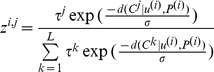
(9)


### Indel Detection Algorithm

Our next task is to use BCV to analyze a sample containing homologous DNA variants, including some with indels. The challenge here is to determine indel positions with respect to a reference sequence from the vocabulary. We introduce the following definitions:

D8. *Main group of DNA variants
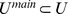
*is a set of variants that align with each other variant from the main group without internal deletions and that represent a sufficient proportion of the mixture (*γ^main^*). The main group is formed as follows:

- All the groups of the DNA variants that align with each other without internal deletions are sorted in order of their weights decreasing.
- The first group in the list, whose weight is less than 60% of the weight of previous group, is found. All the preceding groups are referred as the “head of the list”.
- If there is a single group at the head of the list, it is defined as the main group; otherwise the main group is defined as a group from the head of the list that contains the DNA variant that was generated at the first iteration of the greedy deconvolution algorithm (“strain_1”). This variant is the most similar to the vocabulary sequences. If the “strain_1” is not a member of any group in the head of the list, then the first group (with highest weight) is defined as the main group.

D9. The ***d-*** *shifting pattern* is a set of non-overlapping intervals on the chromatogram sequence corresponding to the optimal partition *B: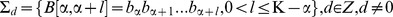,* where ***d*** is a pattern shift size. Intervals of the shifting pattern obey the rule:


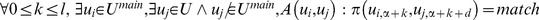
, where *A(u_i_, u_j_)* is the optimal global pairwise alignment of DNA variant sequences.

Ideally, a shifting pattern consists of a single segment. If *d >0*, then the pattern corresponds to an insertion, and if *d <0*, it corresponds to a deletion relative to the main group. We define a shifting pattern *d = 0* as the main group of DNA variants; a value ***d*** is called the *shift size*.

The *beginning* of a shifting pattern is the position on the chromatogram sequence that corresponds to the minimal left coordinate of the pattern’s segments (if reading in the forward direction), or to the maximal right coordinate of the pattern’s segments (if reading in the reverse direction).

The *end* of a shifting pattern is analogously defined. The end of a shifting pattern exists if there is another shifting pattern for the same chromatogram that begins at the 3′ end to the right of the pattern in the appropriate reading direction. Thus, a single shifting pattern for a chromatogram has no end, and the last pattern in the reading direction has no end. This restriction takes into account the fact that peak amplitudes decrease towards the end, so more positions lose minor peaks, making establishing the true positions where a shifting pattern ends unreliable.

D10. The *consensus sequence of a d-shifting pattern* is the sequence of IUPAC symbols 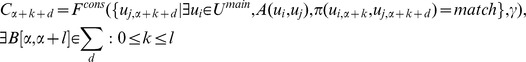, where *F^cons^* is the consensus-building function that projects bases from aligned DNA variants and their fractions on the IUPAC alphabet.

D11. An *indel event* is the deletion or insertion relative to the main group of DNA variants. If the indel event corresponds to the beginning or end of a shifting pattern, we set that *event type* ***d*** equals to the pattern’s shift size. If *d >0*, then the indel event corresponds to an insertion, and if *d <0*, it corresponds to a deletion relative to the main group.

For events corresponding to consecutive patterns, the type is defined as follows. If 2 successive patterns in the chromatogram reading direction have type values of *d1* and *d2*, then the corresponding event has type value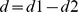.

Additional conditions may be superimposed on the generation of events, e.g, a limit on the maximum distance between consecutive patterns.

For each event, we associate a pair of coordinates (*lpos*, *rpos*) on the alignment, defining an interval in which an indel has occurred; a pair of shift size values for shifting patterns (*d1*, *d2*) that caused the event; and a likelihood (*L*) expressed in terms of alignment weight of profiles corresponding to the main group and the shifting pattern at each event boundary in the window of a given size: 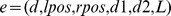.

To introduce the indel detection algorithm, we assume that pairwise global alignments of DNA variants are taken from the global multiple alignment of DNA variants predicted for a sample for all covering chromatograms. We assume that this multiple alignment also contains reference sequences relative to which indel coordinates can be determined.

The pseudo-code for the indel detection algorithm is described below.

**Input.** A multiple alignment of DNA variants *MSA*({*U*}) and DNA variant frequency vectors {*γ*} predicted by the deconvolution algorithm. Here, {…} means a set of elements corresponding to chromatograms of a sample.

**Output.** Indel events with the maximum local likelihood.

Calculations:

1. DNA variants with pairwise alignments that have no internal deletions join separately into groups for each chromatogram.
2. For each chromatogram, determine the main group of DNA variants.
3. Build shifting patterns using definition 9.
4. Generate indel events using definition 11.
5. Estimate event likelihoods.
6. For each chromatogram, separately make groups of events overlapping on alignment coordinates. Only one situation is possible when for an event 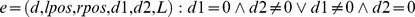, corresponding to the beginning or end of a pattern, there is overlap with another event corresponding to consecutive patterns 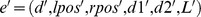; if 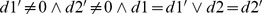 holds, then event *e* is rejected. This rule is based on the Parsimony assumption to obtain the minimum number of events while explaining all shifting patterns for a sample. If it is possible to construct an event corresponding to consistent patterns for a sample’s chromatogram, then this event is always within a set with minimal cardinality.
7. Group all overlapping events of equal type values (***d***) that correspond to different chromatograms.
8. For each group, determine the maximum likelihood event e*, which is included in the final dataset.

### Availability and Future Directions

The source code for the BCV software package is available at the BCV website (http://basecv.sourceforge.net/) for gcc compiler that is available for all UNIX-like (Linux, MAC, Cygwin) platforms. See the website for the examples of the three functionalities and for the documentation. The system requirements are quite moderate: the gcc compiler with installed Boost and GSL libraries are necessary to compile/link; Perl5 is necessary for the scripts to run. The program can work on any standard desktop computer. The executable binary files for Win32 and Linux are also available upon request.

The main limitation of the Sanger sequencing method is the high variation of peak amplitudes in chromatograms, which restricts the accuracy of predicted DNA variants for which the vocabulary has no proper homologue and limits the determination of a sample’s component fractions. Modifying BCV for pyrosequencing data (i.e., not Next Generation Sequencers) that possess a much better signal-to-noise ratio can significantly improve the predictive ability of the method. Another promising direction of the method development is incorporation of *a priori* statistical model of the target DNA if the model is known, e.g. from RNA structure or from a known transcript function, primary or secondary structure.

## Supporting Information

Figure S1 Quality of correspondence of the predicted DNA types to the sequences of known components of the samples. Left tree contains the predicted sequence P (black square) and references (sequences either of the known components or from the dictionary). The right tree contains the reference sequences only (a, b, c, e, f, O). The sequence O is an outgroup for the rooting of the trees. The known components of the mixture are in bold (a, e - black circles). Nodes G_e and G are corresponded nodes (have identical leaf sets with exception of predicted sequences). Node A is a most recent common ancestor (MRCA) for all the known component sequences. Node K is a MRCA of the annotated sequences in G subtree.
(PPT)

Figure S2 Error tolerance of blastn and STAP SSU classifications methods. We built the one dataset of randomly selected 100 sequences that correspond to a PCR fragment of 16S rRNA gene and five datasets obtained by randomly substituting a certain portion of sequence positions in the original dataset with the step of 5%. Sequences in each dataset were classified by the STAP phylogenetic method (see Supplementary Methods S1 for details) and by blastn similarity search against the STAP database. A. Fraction of sequences that kept their original taxonomic assignment in each dataset for both methods – the STAP phylogenetic analysis (Tree2) and blastn. B. Fraction of sequences in each dataset that had blastn refinement of the phylogenetically robust STAP categories: “0” – no differences between the blastn and the STAP taxonomy assignments, “1” – no more than one taxonomy level difference between blastn and the STAP taxonomy assignments (this usually corresponded to blastn refinement up to genera level of the STAP sequence assignment into family taxonomic level).
(XLSX)

Table S1 Primers and PCR programs used in the study
(DOC)

Table S2 A table of the blastn hits in Greengenes database for the BCV predicted sequences. Blastn hits with different taxonomy assignments are shown for one sequence if the assignment scores differ not more than two bits. A. Gastric mucosa sample #95. B. Gastric mucosa sample #97. Read ID is a chromatogram identifier, Cluster ID is an identifier of predicted sequence in BCV output file. Identity shows the percent identity for a blastn hit. Accession is an accession number of a hit sequence in the Greengenes. ProkMSAname column contains the names of the sequence source organisms. Greengenes taxonomy is the taxonomy category that contains the blastn hit sequence in the database. STAP classification shows the taxonomy category, where the predicted sequence has been assigned by STAP.
(DOC)

Table S3 BCV indel calling accuracy (9 b.p. deletion). BCV was tested on the dataset provided in [27] study. The chromatograms for two component mixtures have been processed by BCV. The portion of a variant with deletion in a mixture is specified in the sample name. Each mixture was sequenced in 3 replicates marked by v1–v3, e.g. 15_v3 - the variant with deletion comprises 15% of the mixture and the third replicate has been sequenced. Table rows correspond to the deletions that are predicted by BCV. The predicted indel sizes, location on the reference sequence (ref:loc), location relative to the annotated indel (loc:error) are also shown. The column “primer” contains the names of the sequencing primers. The (!) sign marks the 9 b. p. indel predictions that BCV reports as having the maximal likelihood for the corresponding sample. Notes: 1. Cloned mtDNA fragments occupy positions 101. 367 of the reference sequence. 2. 249–259 - annotated position of the 9 bp deletion [27]. Tandem repeat (CCCCCTCTA)2 is located in positions 250–267, thus the deletion (249,259) is indistingushable of the 9 bp delition (258–268). 3. Indels of length 1–2 bp are likely to be artificial and are ignored in subsequent analysis.
(XLS)

Table S4 BCV indel calling accuracy (51 b.p. deletion). BCV was tested on the dataset provided in [27] study. The chromatograms for two component mixtures has been processed by BCV. The portion of the variant with indel is specified in the sample name. Each mixture was sequenced in 3 replicates marked by v1–v3, e.g. v3_15 - the deletion variant comprizes 15% of the mixture and the third replicate has been sequenced. In the table, rows are deletions predicted by BCV. The predicted indel sizes, locations on the reference sequence (ref:loc), locations relative the annotated indel (loc:error) are shown. The “n.d.” means indel is not detected. Notes: 1. Cloned mtDNA fragments occupy positions 1–722 of the reference sequence. 2. 297–348 - annotated position of the 51 bp deletion [27]. The major variant in the mixture has unannotated insertion of 1 bp within (C)5 repeat in positions 311.315, thus the 51 bp deletion in the minor variant corresponds to 50 bp deletion relative the reference sequence (NC_012920). The motif (CCAAACCCCC) is located in positions 298.306 and in 348.356, thus the deletion (297,348) is indistingushable of the 51 bp delition (306–357). 3. Indels of length 1–2 bp are likely to be artificial and are ignored in subsequent analysis.
(XLS)

Table S5 Results of the BLAST search of BCV-predicted DNA variants in Greengenes database for RipSeq example chromatograms. The BCV mixture deconvolution analysis was applied to usage examples available for RipSeq web server (https://www.ripseq.com/login/login.aspx Accessed 20 Dec. 2012). For each chromatogram the corresponding RipSeq prediction (RipSeq pred.) is shown. Sequences predicted by BCV for each chromatogram (their names are shown in the “BCV pred.” column) that have the expected portion in a mixture higher than 5% are searched by BLAST versus Greengenes database (http://greengenes.lbl.gov/cgi-bin/nph-index.cgi Accessed 20 Dec. 2012) with default parameters. The score of the best hit, identity, alignment length, accession number and the organism name are shown for each BCV prediction. Blast hits those scores are fewer by no more than two bits than the best hit score also are shown. For each genus only one prediction with the maximum score allowed. Hits with identity less than 80% were ignored. The predicted species for each chromatogram are in bold. *The *Peptostreptococcus micros* is a basonym for the *Parvimonas micra*.
(XLS)

Methods S1 (DOC)
