# Supplementary figures and images for: Base-Calling Algorithm with Vocabulary (BCV) Method for Analyzing Population Sequencing Chromatograms
Source: PLoS One. 2013 Jan 28;8(1):e54835. doi: 10.1371/journal.pone.0054835 (PMC3557274; doi:10.1371/journal.pone.0054835)

## Slide 1
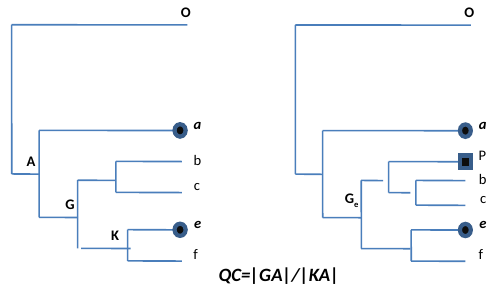

O
a
b
A
c
G
e
K
f
O
a
P
b
Ge
c
e
f
QC=|GA|/|KA|

Supplement: Figure S1 — Quality of correspondence of the predicted DNA types to the sequences of known components of the samples. Left tree contains the predicted sequence P (black square) and references (sequences either of the known components or from the dictionary). The right tree contains the reference sequences only (a, b, c, e, f, O). The sequence O is an outgroup for the rooting of the trees. The known components of the mixture are in bold (a, e - black circles). Nodes G_e and G are corresponded nodes (have identical leaf sets with exception of predicted sequences). Node A is a most recent common ancestor (MRCA) for all the known component sequences. Node K is a MRCA of the annotated sequences in G subtree. (PPT) [file pone.0054835.s001.ppt]
