# Supplementary material for: Base-Calling Algorithm with Vocabulary (BCV) Method for Analyzing Population Sequencing Chromatograms
Source: PLoS One. 2013 Jan 28;8(1):e54835. doi: 10.1371/journal.pone.0054835 (PMC3557274; doi:10.1371/journal.pone.0054835)
Supplement: Table S1 — Primers and PCR programs used in the study (DOC) [file pone.0054835.s003.doc]

**Supplementary Table S1. Primers and PCR programs used in the study**

| **Sample** | **Primer** | **Region** | **Sequence (5′→3′)** | **PCR program** |
| --- | --- | --- | --- | --- |
| ***M. tuberculosis*** | PR9* | *PncA* gene | According to [Sekiguchi07] | According to [Sekiguchi07] |
|  | PR10* |  |  |  |
| **HIV** | first round of nested RT-PCR: hiv-pf1 | *Gag* gene (p1, p6) and *PR-*coding region | AGG AAA ARG GGC TGT TGG AAA TGT | first round of nested RT-PCR:  1. 45°C - 30 min; 2. 95°C - 5 min;  3. 30 cycles of: 95°C - 30 s 50°C - 30 s 72°C - 30 s;  4. 72°C - 1 min |
|  | first round of nested RT-PCR: hiv-pr1 |  | GCT TTT ATT TTK TCT TCT GTC AAT GGC CAA |  |
|  | second round of nested PCR: hiv-pf2* |  | CCA AAT GAA AGA ITG YAC TGA RAG ACA GGC T | second round of nested PCR:  1. 95°C - 5 min;  2. 30 cycles of: 95°C - 30 s 56°C - 30 s 72°C - 30 s;  3. 72°C - 1 min |
|  | second round of nested PCR: hiv-pf2* |  | GGC CAT TGT TTA ACY TTT GGI CCA TCC AT |  |
| **HBV** | hbv-rt-F* | *P*-gene | gCA gTC CCC AAC CTC CAA TCA CT | 1. 95°C - 5 min;  2. 50 cycles of:  95°C - 30 s 62°C - 30 s 72°C - 50 s;  3. 72°C - 1 min |
|  | hbv-rt-R* |  | Agg gTT gCg TCA gCA AAC ACT Tg |  |
|  | hbv-rt-S** |  | ggA CCA TgC ARA ACC TgC ACg ACT CCT |  |
| **HAV** | AP1* | *2C* gene | CTA TGA TGT TTG GAT TCC ATC ATT CTG T | 1. 95°C - 5 min;  2. 42 cycles of:  95°C - 30 s 54°C - 30 s 72°C - 50 s;  3. 72°C - 1 min |
|  | AR3* |  | CTC AGG CTC AAC ACC ATA GTG TTT ACA |  |
| **HDV** | hdv-1G* | *HDAg* coding region | CAG GTC GGA CCG CGR GGA GGT GGA GAT | 1. 95°C - 5 min;  2. 42 cycles of: 95°C - 30 s 67°C - 30 s 72°C - 30 s;  3. 72°C - 1 min |
|  | hdv-2G* |  | GAT CAC CGA MGA AGG AAG GCC CTS GAG AA |  |
| **Gastric mucosa samples** | Un161* | *16S rRNA* gene | AGT GGC GIA CGG GTG AGT AAC | 1. 95°C - 5 min;  2. 30 cycles of: 95°C - 10 s 60°C - 20 s 72°C - 20 s;  3. 72°C - 5 min |
|  | Un162* |  | CAT CTC ACG ACA CGA GCT GAC GA |  |

*- primer was used in both PCR and the sequencing reaction

** - primer was used only in the sequencing reaction

Sekiguchi,J., Miyoshi-Akiyama,T., Augustynowicz-Kopeć,E., Zwolska,Z., Kirikae,F., Toyota,E., Kobayashi,I., Morita,K., Kudo,K., Kato,S., et al. (2007) Detection of multidrug resistance in Mycobacterium tuberculosis. *J. Clin. Microbiol.*, **45**, 179–192.
