# Supplementary material for: Base-Calling Algorithm with Vocabulary (BCV) Method for Analyzing Population Sequencing Chromatograms
Source: PLoS One. 2013 Jan 28;8(1):e54835. doi: 10.1371/journal.pone.0054835 (PMC3557274; doi:10.1371/journal.pone.0054835)
Supplement: Table S2 — A table of the blastn hits in Greengenes database for the BCV predicted sequences. Blastn hits with different taxonomy assignments are shown for one sequence if the assignment scores differ not more than two bits. A. Gastric mucosa sample #95. B. Gastric mucosa sample #97. Read ID is a chromatogram identifier, Cluster ID is an identifier of predicted sequence in BCV output file. Identity shows the percent identity for a blastn hit. Accession is an accession number of a hit sequence in the Greengenes. ProkMSAname column contains the names of the sequence source organisms. Greengenes taxonomy is the taxonomy category that contains the blastn hit sequence in the database. STAP classification shows the taxonomy category, where the predicted sequence has been assigned by STAP. (DOC) [file pone.0054835.s004.doc]

**Supplementary Table S2**. A table of the blastn hits in Greengenes database for the BCV predicted sequences.

A

| Read ID (D/R) | Cluster ID | Score | Identity % | Match length | Accession | prokMSAname | Greengenes taxonomy | STAP classification |
| --- | --- | --- | --- | --- | --- | --- | --- | --- |
| 161  (D) | 1 | 396 | 95.87 | 484 | EF108448.1 | Veillonella sp. str. NVG 24cf | s__Veillonella parvula | Bacteria|Firmicutes|Acidaminococcaceae|Dialister|Veillonella |
|  | 12 | 133 | 85.96 | 235 | ACWY01000141.1 | Prevotella melaninogenica str. D18 | Unclassified | Bacteria\|Bacteroidetes\|Bacteroidales\|Bacteroidaceae\|Prevotellaceae |
| 162  (R) | 1 | 509 | 97.97 | 543 | AY995770.1 | Veillonella dispar str. ATCC 17748 | s__Veillonella dispar | Bacteria|Firmicutes|Acidaminococcaceae|Dialister|Veillonella |
|  | 9 | 325 | 95.74 | 376 | AJ626902.1 | Enterococcus saccharominimus str. LMG 21727 | g__Enterococcus | Bacteria\|Firmicutes\|Bacilli\|Lactobacillales\|Unclassified |
| 16Sq  (R) | 1 | 218 | 84.68 | 359 | FQ312002.1 | Haemophilus parainfluenzae str. T3T1 | Unclassified | Bacteria|Proteobacteria|Gammaproteobacteria|Pasteurellaceae|Unclassified |
|  | 3 | 191 | 90.87 | 263 | AF287813.1 | Leptotrichia sp. oral strain str. FAC5 | g__Leptotrichia | Bacteria\|Fusobacteria\|Leptotrichales\|Leptotrichiaceae |
|  | 3 | 189 | 90.49 | 263 | NR_025647.1 | Leptotrichia hofstadii str. LB23; CCUG 47504 | g__Leptotrichia |  |
|  | 2 | 227 | 86.10 | 374 | NZ_AABF02000026.1 | Fusobacterium nucleatum subsp. vincentii str. ATCC 49256 | g__Fusobacterium\| | Bacteria\|Fusobacteria\|Fusobacteriaceae\|Fusobacterium |

B

| Read ID (D/R) | Cluster ID | Score | Identity % | Match length | Accession | prokMSAname | Greengenes tax | STAP classification |
| --- | --- | --- | --- | --- | --- | --- | --- | --- |
| 161  (D) | 1 | 473 | 97.96 | 539 | DQ202377.1 | Helicobacter pylori str. DM1A | s__Helicobacter pylori | Bacteria|Proteobacteria|Epsilonproteobacteria|Helicobacterales|Helicobacteraceae|Helicobacter|Helicobacter_pylori|Helicobacter_acinonychis |
|  | 8 | 384 | 91.34 | 508 | AF110272.1 | Sarcina ventriculi | g__Sarcina | Bacteria\|Firmicutes\|Clostridia\|Clostridiales\|Clostridiaceae\|Unclassified |
| 162  (R) | 1 | 511 | 98.53 | 545 | NC_011333.1 | Helicobacter pylori str. G27 | s__Helicobacter pylori | Bacteria|Proteobacteria|Epsilonproteobacteria|Helicobacterales|Helicobacteraceae|Helicobacter|Helicobacter_pylori|Helicobacter_acinonychis |
|  | 2 | 360 | 95.07 | 426 | X76649.1 | Sarcina ventriculi str. DSM286 | g__Sarcina | Bacteria\|Firmicutes\|Clostridia\|Clostridiales\|Clostridiaceae\|Unclassified |
| 16Sq  (R) | 1 | 416 | 99.31 | 434 | X76649.1 | Sarcina ventriculi str. DSM286 | g__Sarcina | Bacteria|Firmicutes|Clostridia|Clostridiales|Clostridiaceae|Unclassified |

Blastn hits with different taxonomy assignments are shown for one sequence if the assignment scores differ not more than two bits. A. Gastric mucosa sample #95. B. Gastric mucosa sample #97. Read ID is a chromatogram identifier, Cluster ID is an identifier of predicted sequence in BCV output file. Identity shows the percent identity for a blastn hit. Accession is an accession number of a hit sequence in the Greengenes. ProkMSAname column contains the names of the sequence source organisms. Greengenes taxonomy is the taxonomy category that contains the blastn hit sequence in the database. STAP classification shows the taxonomy category, where the predicted sequence has been assigned by STAP.
