# Supplementary material for: Base-Calling Algorithm with Vocabulary (BCV) Method for Analyzing Population Sequencing Chromatograms
Source: PLoS One. 2013 Jan 28;8(1):e54835. doi: 10.1371/journal.pone.0054835 (PMC3557274; doi:10.1371/journal.pone.0054835)
Supplement: Methods S1 — (DOC) [file pone.0054835.s008.doc]

## *Base-calling Algorithm with Vocabulary (BCV) Method for Analyzing Population Sequencing Chromatograms*

Yuri S. Fantin, Alexey D. Neverov, Alexander V. Favorov, Maria V. Alvarez-Figueroa, Svetlana I. Braslavskaya, Maria A. Gordukova, Inga V. Karandashova, Konstantin V. Kuleshov, Anna I. Myznikova, Maya S. Polishchuk, Denis A. Reshetov, Yana A. Voiciehovskaya, Andrei A. Mironov and Vladimir P. Chulanov

## Supplementary Methods

**Nucleic acids methods**

*M. tuberculosis* DNA, HBV DNA from the df7 sample, HBV DNA extracted from BV1, and DNA from the human gastric mucosa samples were used for PCR amplification. Extracted HAV RNA and HDV RNA were reverse-transcribed, and the cDNA was amplified by PCR (see below). Extracted HIV RNA from GEN014DR.01A sample was used for first-round reverse transcription PCR (RT-PCR) amplification, after which the RT-PCR product was used for a second round of nested PCR. The amplification products of the df7 sample, *M. tuberculosis*, HAV, HIV, and HDV were analyzed by sequencing.

Bacterial and viral DNA or RNA (except HIV RNA) were isolated using the RIBO-Prep kit (CRIE, Russia), and plasmid DNA was isolated using the Axyprep® Plasmid Miniprep Kit (Axygen, Union City, CA, USA) according to manufacturers' protocols. DNA from the gastric mucosa samples was extracted using RIBO-Prep from homogenized biopsies, with nucleic acid-free water in the elution step (RT-PCR Grade Water, Ambion, Grand Island, NY, USA). HIV RNA was isolated with the RIBO-Sorb kit (CRIE, Russia).

Reverse transcription of HAV RNA and HDV RNA was performed using the REVERTA-L kit (CRIE, Russia) according to the manufacturer's protocol.

PCR amplification of *M. tuberculosis* DNA was performed using AmpliSens® PCR Hot Start (2,4 mM Mg2+;CRIE, Russia). PCR amplification of HAV cDNA, HDV cDNA, HBV DNA, and DNA from gastric mucosa samples was performed using AmpliSens® PCR (3,0 mM Mg2+;CRIE, Russia). RT-PCR and nested PCR of HIV RNA was performed using the AmpliSens® HIV-Genotype-Eph kit (CRIE, Russia) according to the manufacturer’s instruction. Primers and programs for PCR amplification are shown in Table S1.

*Cloning and sequencing*. PCR products of following samples were cloned and selected numbers of colonies (in brackets) were sequenced:

HIV: GEN014DR.01A (9);

HBV: BV1(10);

*M. tuberculosis*: #2687 (10), MS41 (9), #11042 (9);

Gastric mucosa samples: #95 (15), #97 (10).

pGEM®-T Vector System (Promega, Madison, WI, USA) was used to clone the PCR-amplified fragments. Sequencing of the PCR products was performed using the BigDye® Terminator v1.1 Cycle Sequencing Kit and the automatic sequencer ABI-PRISM 3100 (both from Applied Biosystems, Grand Island, NY, USA) according to the manufacturer's protocols.

**Algorithm testing**

*Trimming of primer and vector sequences.* The BCV indel detection and deconvolution functionalities can be used for chromatogram analysis if sequences in a vocabulary cover a target locus (or the most part of it). If chromatogram sequences contain parts of vector sequences, the positions of the target locus should be specified for each chromatogram in the project file that is input for the *bcv_run.pl* script. Pregap4 [1] from the STADEN package [2,3] can be used for detecting vector contaminations in chromatograms. BCV automatically extracts positions of the target locus from pregap4 output if the path to the folder with *.exp files has been set in the BCV project file (see BCV User Manual).

We used chromatograms from the study [4] for estimating the BCV minor indel detection limit. Chromatograms in the dataset had large parts of the cloning vector sequences. We used pregap4 for clipping vector sequences and preparing datasets for the BCV analysis.

*Assessing base-calling accuracy*. The quality of nucleotide sequence predictions was assessed on two sets of direct sequencing traces: the HAV (79 traces) and the HDV set (39 traces). An annotated sequence for each trace was obtained by assembling two chromatograms obtained in both directions (ContigExspress, [5]). The length of annotated sequences was 700 bp in the HAV set and 450 bp in the HDV set; the average number of trace positions was 680 in the HAV set and 410 in the HDV set.

Hepatitis A is an acute disease, and the HAV population is usually genetically homogeneous, without degenerate sequence positions. Samples from the HDV set are mostly obtained from chronically infected patients, and HDV sequences show approximately 3–14% degeneracy.

BCV predictions were compared with results obtained by three other base-callers: ABI Basecaller 3100 [6], TraceTuner v. 3.01 [7], and PolyScan [8]. Chromatogram sequences for PolyScan were generated from the corresponding BQS files (see BCV software data flow) taking peaks with probabilities higher than 0.3 to be true. For each chromatogram, the multiple alignment of the four predicted sequences and an annotated sequence was built by Muscle [9]. Up to four different nucleotides were permitted in each position of a predicted sequence. IUPAC encoding was used to represent degenerate sequence positions. For each pairwise sub-alignment of a predicted and an annotated sequence, the following events were considered: a match event if the IUPAC symbols in both sequences were identical; a similarity event if the 2 sets of IUPAC symbols have a non-empty intersection; and a mismatch event in the case of an empty intersection or an indel event. The standard measures of accuracy (i.e., sensitivity, specificity, and identity) were calculated on each dataset.

*Calculating the quality of correspondence between predicted and actual sample components.*

The actual DNA variants in our samples were known in advance, and we refer to them as sequences of annotation. We assessed the correspondence of the set of predicted sequences to the sequences of annotation relative to BCV vocabulary. Indeed, the vocabulary is supposed to represent the possible genetics variations in a sequenced genomic region, so the phylogenetic tree of vocabulary sequences and the sequences of annotation (the reference tree) give us a scale to evaluate the correspondence of predictions to annotation.

Let us consider two phylogenetic trees, with the sequences as the leaves: the reference tree and the tree built of the reference tree sequences and the predicted sequences (estimating tree). We believe that both these trees were rooted at the same (outgroup) sequence and grow down.

A pair of nodes from the two trees is referred to as corresponded nodes if: a) the sets of leaves excluding predicted sequences that belong to these nodes' subtrees are the same; and b) predicted sequences in the estimating tree are not direct descendants of these nodes. Condition (b) is for one-to-one correspondence between internal nodes of the reference tree and some internal nodes of the estimating tree (Fig. S1). Let us consider a predicted sequence and the corresponding closest annotated sequence. If the most recent common ancestor (MRCA) of these sequences on the estimating tree meets condition (b), we denote it as G_e. Otherwise, we move upwards until we obtain a node that meets (b) and denote it G_e. Now we consider node G that corresponds to G_e on the reference tree, or, in simpler terms, that represents the closest ancestor of the predicted sequence on the reference tree. Now we intend to evaluate how close it is to one of the annotated sequences compared to others in the scale of the tree. This is supposed to measure the quality of correspondence (QC) of the predicted sequence to the set of sequences of annotation. The MRCA node of all annotated sequences was denoted as A in the reference tree. With a single annotated sequence, the QC = 1 if G coincides with the ancestor of the node A or 0 otherwise. If there are several annotated sequences, and node G coincides with A or is situated upwards from A on the reference tree, then the QC = 0 for this predicted sequence. If G is below A, the MRCA of the annotated sequences in the subtree of node G is denoted as K. Thus, for that predicted sequence, QC = |GA|/|KA|, where |GA| and |KA| are the distances between the nodes. K can be lower than G or can coincide with G, so 0 ≤ QC ≤ 1.

For each predicted DNA variant, BCV calculated the expected value of its share in a sample mixture, so the QC for the set of predicted sequences is a convolution (weighted average) of the QCs for the individual sequences.

When calculating the QC, multiple alignments were prepared using Muscle software [9]. Phylogenetic analysis was performed by the method of minimum evolution FastME [10], and the distance matrix was calculated using dnadist [11]. The evolutionary model F84 + G (α = 0.41, Tr / Tv = 1.52) that we used was obtained from aligning the sequences in dictionary HBVRT by Modeltest from the MEGA5 package [12].

*Classifications of ss-rRNA sequence collections.* DNA was isolated from two gastric mucosa biopsy samples. A fragment of the 16S rRNA gene approximately 840 bp long was amplified by PCR. The PCR product was sequenced from three primers in both directions. Then the samples were characterised by cloning and sequencing of selected clones.

To classify the ss-rRNA sequences after the cloning step, that is, to assign the sequences to bacterial taxonomy, we used a method based on STAP [13] and the RDP Classifier [14].

For BCV-predicted sequences, those expected frequencies were greater than 5% and lengths were at least 50% of chromatogram sequence length, we used the STAP-based algorithm because it tolerates sequencing errors (random substitutions) that can widely occur in the BCV results, especially in low-quality chromatograms (see Errors in the predicted BCV sequences for 16S rRNA direct sequencing chromatograms).

We modified STAP as follows. The original version used the maximum likelihood method PhyML [15] for constructing a phylogenetic tree and assigned taxonomy of the closest neighbour to a sequence. We used SH-like aLRT with a threshold of 80% to determine the reliability of the phylogenetic tree branches as recommended by the PhyML documentation. Then we assigned to the sequence the first reliable taxonomy category (tree node) on the way between the original sequence and the outgroup.

In general, RDP provides more detailed classification than STAP. Some clone sequences that STAP classified only up to the family level were classified up to the genus by RDP; these included family *Prevotellaceae* genus *Prevotella*, family *Pasteurellaceae* genus *Haemophilus*,and family *Clostridiaceae* genus *Sarcina*. The differences occurred mostly due to the specificity of the STAP database. Despite this, STAP classification provides an undeniable advantage in terms of reliability of taxonomic categories to which sequences were assigned, especially for sequences with high error rate.

*Errors in the predicted BCV sequences for 16S rRNA direct sequencing chromatograms.* To estimate the error level in predicted BCV 16S rRNA sequences that could be tolerated by the STAP classification method, we constructed simulations. A set of 100 subsequences of 16S rRNA gene fragment from the BCV vocabulary that corresponded to this study's PCR primers (see tab. S1) were randomly selected and then classified by STAP. Then, five sets with error levels from 5% up to 25% with 5% increments were constructed from the original sequences by random point mutations. These “noised” sets were also classified by STAP. Figure S2A shows the proportion of the sequences in each set that conserved the original taxonomy assignment. The two curves correspond to two different classification methods: using Blastn [16] best match, and using STAP phylogenetic analysis (Tree2). We noticed that if the number of changed positions did not exceed 15% of a sequence, then about 92% and 98% of sequences were classified by STAP and Blastn, correspondingly, into the original categories. At the higher noise levels the accuracy of the Blastn classification dropped more sharply than the accuracy of the STAP, e.g. if portion of changed positions exceeds 20% than about 90% of the dataset were addressed into original category by the phylogenetic analysis and only 86% by the similarity search. More than 90% and 80% of Blastn hits fell just into or no more than one level upper from the assigned STAP taxonomic category (which mostly corresponds to genus or family level) if an error level in sequences did not exceed 15% and 20% correspondingly (fig. S2B).

If substitutions in the sequences are not random and are localized in sites that determine phylogeny, then phylogenetic analysis would not provide acceptable accuracy of classification. The range of 16S rRNA sequence similarities that was usually related to a species was over 97%, and to a genus and to a phylum was > 95% and > 80%, respectively [17]. We simulated random replacements in phylogenetically-informative sites by substituting nucleotides in each sequence with different nucleotides from a randomly chosen pairwise alignment of the original sequence, with similarity levels below 80% (i.e., a sequence from a different phylum). Even if a share of mutated sites was only 5%, only ~75% of the sequences were accurately classified by phylogenetic analysis, while Blastn classified approximately 98% of the sequences. When half of the informative sites were changed (10% replacements of total sites), STAP could identify approximately ~30% of sequences, while Blastn correctly classified ~70% sequences.

If there were no contradictions in taxonomy assignment between the STAP phylogenetic analysis and the Blastn similarity search, and the STAP category was not very broad (e.g. genus or family), the Blastn could be considered a refinement method for a phylogenetically robust STAP category that allows us to assume a mostly random nature of errors in a predicted sequence.

## References

1. Bonfield JK, Staden R (1996) Experiment files and their application during large-scale sequencing projects. DNA Seq 6: 109–117.

2. Staden R (1996) The Staden sequence analysis package. Mol Biotechnol 5: 233–241.

3. Staden R, Beal KF, Bonfield JK (2000) The Staden package, 1998. Methods Mol Biol 132: 115–130.

4. Zhidkov I, Cohen R, Geifman N, Mishmar D, Rubin E (2011) CHILD: a new tool for detecting low-abundance insertions and deletions in standard sequence traces. Nucleic Acids Res 39: e47. doi:10.1093/nar/gkq1354.

5. Lu G, Moriyama EN (2004) Vector NTI, a balanced all-in-one sequence analysis suite. Brief Bioinformatics 5: 378–388.

6. Hagemann TL, Kwan S-P (1999) ABI Sequencing Analysis: Manipulation of Sequence Data from the ABI DNA Sequencer. MB 13: 137–152. doi:10.1385/MB:13:2:137.

7. Denisov GA, Arehart AB, Curtin MD (2004) A system and method for improving the accuracy of DNA sequencing and error probability estimation through application of a mathematical model to the analysis of electropherograms. US Patent 6681186.

8. Chen K, McLellan MD, Ding L, Wendl MC, Kasai Y, et al. (2007) PolyScan: An automatic indel and SNP detection approach to the analysis of human resequencing data. Genome Research 17: 659–666. doi:10.1101/gr.6151507.

9. Edgar RC (2004) MUSCLE: multiple sequence alignment with high accuracy and high throughput. Nucleic Acids Res 32: 1792–1797. doi:10.1093/nar/gkh340.

10. Desper R, Gascuel O (2002) Fast and accurate phylogeny reconstruction algorithms based on the minimum-evolution principle. J Comput Biol 9: 687–705. doi:10.1089/106652702761034136.

11. Felsenstein J (1981) Evolutionary trees from DNA sequences: a maximum likelihood approach. J Mol Evol 17: 368–376.

12. Tamura K, Peterson D, Peterson N, Stecher G, Nei M, et al. (2011) MEGA5: molecular evolutionary genetics analysis using maximum likelihood, evolutionary distance, and maximum parsimony methods. Mol Biol Evol 28: 2731–2739. doi:10.1093/molbev/msr121.

13. Wu D, Hartman A, Ward N, Eisen JA (2008) An Automated Phylogenetic Tree-Based Small Subunit rRNA Taxonomy and Alignment Pipeline (STAP). PLoS ONE 3: e2566. doi:10.1371/journal.pone.0002566.

14. Wang Q, Garrity GM, Tiedje JM, Cole JR (2007) Naive Bayesian classifier for rapid assignment of rRNA sequences into the new bacterial taxonomy. Appl Environ Microbiol 73: 5261–5267. doi:10.1128/AEM.00062-07.

15. Guindon S, Dufayard J-F, Lefort V, Anisimova M, Hordijk W, et al. (2010) New algorithms and methods to estimate maximum-likelihood phylogenies: assessing the performance of PhyML 3.0. Syst Biol 59: 307–321. doi:10.1093/sysbio/syq010.

16. Altschul SF, Madden TL, Schäffer AA, Zhang J, Zhang Z, et al. (1997) Gapped BLAST and PSI-BLAST: a new generation of protein database search programs. Nucleic Acids Res 25: 3389–3402.

17. Schloss PD, Handelsman J (2005) Introducing DOTUR, a computer program for defining operational taxonomic units and estimating species richness. Appl Environ Microbiol 71: 1501–1506. doi:10.1128/AEM.71.3.1501-1506.2005.
